# Effect of Gold Nanoparticles and Coexisting Acetonitrile Solvent on the Structure of Bovine Serum Albumin

**DOI:** 10.1021/acsomega.5c05552

**Published:** 2025-10-10

**Authors:** Samal Kaumbekova, Kyoko Omata, Ryo Nagasawa, Masakazu Umezawa

**Affiliations:** † Department of Medical and Robotic Engineering Design, Faculty of Advanced Engineering, 378550 Tokyo University of Science, 6-3-1 Niijuku, Katsushika, Tokyo 125-8585, Japan; ‡ Department of Chemistry, Faculty of Natural Sciences, L. N. Gumilyov Eurasian National University, Astana 010000, Kazakhstan; § Department of Materials Science and Technology, Graduate School of Advanced Engineering, 378550 Tokyo University of Science, 6-3-1 Niijuku, Katsushika, Tokyo 125-8585, Japan

## Abstract

In the design of biocompatible protein-based drug delivery systems (DDS), the protein structural stability is important for its proper function. While organic solvents widely used during the preparation of protein-based DDS might induce structural changes in the proteins, the presence of nanoparticles (NPs) might additionally alter the protein structure. Although previous studies have reported various effects of NPs on protein structure in various environments, there is a lack of understanding of the effect of the coexisting organic solvent environment on designing a biocompatible DDS. In this study, we investigated the effect of 5 nm gold NPs (AuNPs) on albumin structural stability in the presence of organic solvent, such as acetonitrile (ACN). Bovine serum albumin (BSA) was chosen as an albumin—a transport protein model with high abundance in the blood, high stability, and possible applications in DDS. In addition, AuNPs were used in this study due to their possible applications in DDS, therapeutics, sensing, imaging, and biotechnology. ACN was chosen as an organic solvent with wide applications in DDS and the pharmaceutical industry, and its concentration was varied to investigate the effect of different solvent polarities. Circular dichroism spectroscopy showed that the presence of AuNPs caused partial unfolding of the BSA structure, with increased hydrodynamic diameter distribution in the presence of 0%–15% vol. ACN. While the most prominent effect was observed at 15% ACN, such results were associated with enhanced protein-ACN interactions in the presence of AuNPs, as revealed by MD simulations. Furthermore, while ACN molecules strongly interacted with the protein subdomains bound to AuNPs, water molecules had a higher propensity to interact with the subdomains located far from the NP. Overall, such distinct interactions of protein subdomains with water and coexisting organic solvents may enhance the protein’s structural changes and unfolding in the presence of NPs, leading to a better design of drug loading into proteins.

## Graphical abstract

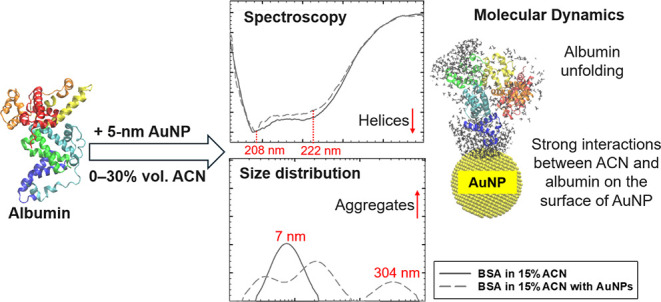

## 1 Introduction

The drug delivery system (DDS) aims to efficiently encapsulate and safely distribute biomolecules, such as proteins, gene therapy materials, and drugs with low solubility, to targets within living tissue.^1^ To prevent the toxicological effects of nanocarriers, protein-based DDS have gained interest as biocompatible colloidal nanomaterials for drug delivery.^2^ One of the traditional techniques for creating protein-based drug carriers with consistent sizes is the desolvation procedure, carried out in the presence of organic solvents, known as desolvating agents.^3^ While proteins might be used as an alternative biocompatible DDS, they might lose their structural stability and unfold due to cosolvent-induced protein denaturation.^4–6^ Furthermore, the aggregation of proteins into amyloid-like structures might affect their binding affinity to drugs and other metabolites.^7^ Moreover, while low drug concentration induces protein stabilization (folding), high drug concentration brings a partial protein destabilization (unfolding), as was experimentally observed in the drug–protein complexes with human serum albumin (HSA).^8^ In addition, the reduction in α-helix and increase in β-structure in HSA were revealed in the drug–protein complexes, associated with the partial unfolding of HSA during drug loading.^9^

Considering the importance of protein structural stability for its proper function and to avoid nanotoxicity,^10^ a potential pathway to improve the biocompatibility of DDS is to stabilize protein conformation via conjugation with nanoparticles (NPs), forming a stable protein-NP conjugate, known as protein corona (PC).^11^ Furthermore, numerous literature studies show a growing interest in the applications of PC-based DDS due to their advantages over NP-based DDS, including improved biocompatibility and circulation time.^12^ Experimental studies showed that PC can inhibit the toxicity of NP-based DDS by inhibiting the uptake of NPs into the cells.^13^ For example, coating gold nanorods with the protein keratin reduces cytotoxicity and increases the colloid stability of NPs.^14^ The cytotoxicity of silver-based NPs was reduced by coating with bovine lactoferrin protein without any significant effect on the protein conformation and structural stability.^15^ Moreover, recent experimental studies investigated the complexation of doxorubicin (DOX), bovine serum albumin (BSA), and citrate-stabilized gold nanoparticles (AuNPs), revealing a high binding ability of BSA to AuNPs and high adsorption of drug molecules on AuNPs, affecting the dynamics of the DDS formation.^16^

The interactions between AuNPs and proteins are of high interest because of the potential uses of AuNPs in biotechnology, imaging, sensing, and DDS.^17^ A study on the effect of AuNPs on native and denatured forms of BSA showed the BSA protein coating on the surface of AuNPs for both native and denatured forms of protein, representing the BSA stabilized AuNPs.^18^ Although the early studies reported the formation of PC after the interaction of proteins with AuNPs,^19^ recent studies highlighted the importance of the protein concentration in the formation of a stable PC.^20,21^ Furthermore, the loss of helical structures in the BSA was observed at elevated particle concentrations of citrate-stabilized AuNPs.^19^ The AuNPs might alter the secondary structure of proteins and cause partial protein unfolding, allowing the solvent to access the hydrophobic cavity of the protein, affecting the polarity and microenvironment around Trp-212.^22^ The study on the bioconjugation of BSA and AuNPs in the presence of cationic surfactants showed that upon bioconjugation, the Trp amino acid residues would be exposed to a relatively more polar environment in bioconjugated unfolded protein.^23^ A recent study on the effect of AuNPs with different morphologies (nanospheres, nanoflowers, and nanorods) on HSA also showed that all three types of NPs altered the polarity of the hydrophilic environment around the Trp-residues in HSA and reduced helical content in the protein structure.^24^

A recent study on the effect of surface coating of AuNPs showed that capping NPs with small ligands promoted nonspecific protein adsorption, suggesting that small molecules enhanced the homogeneous solubility of the NPs in water via electrostatic repulsions.^25^ In addition, surface functionalization of AuNPs by curcumin and turmeric extract resulted in a mild decrease in the helical content of HSA, associated with a high surface curvature of NPs with small size (the average AuNPs size of around 12.9 nm, as reported by DLS analysis).^26^ Moreover, recent studies showed the partial unfolding of the BSA structure in the presence of gold nanoclusters (Au-NCs) and a coexisting alkaline environment.^27^ Furthermore, induced changes and decreased helical content and increased β-turns in the BSA conformation were observed in the BSA-AuNP conjugates at extreme pH values, as was revealed by spectroscopic studies.^28–30^ In addition, the loss of the secondary structure of bovine serum albumin (BSA) was observed in the presence of AuNPs and coexisting salt environment, as was revealed by spectroscopical analysis and molecular dynamics (MD) simulations.^31^

Although recent experimental studies have revealed the altered effect of AuNPs on the protein structure in the coexisting alkaline and salt environments, there is a lack of understanding of the coexisting organic solvent environments to design a biocompatible DDS. Herein, we investigated the effect of 5 nm AuNPs on the structural stability of albumin in the presence of organic solvent at various solvent polarities. While albumin is the most abundant transport protein in the blood, with high stability and possible applications in DDS,^32^ BSA was chosen as an albumin protein model. In addition, AuNPs were used in this study due to their possible applications in DDS, therapeutics, sensing, imaging, and biotechnology.^17^ Acetonitrile (ACN) was chosen as an organic solvent with wide applications in DDS and the pharmaceutical industry.^33,34^ Although DDS is intended for use in physiological aqueous environments, organic solvents such as ACN are commonly encountered during various stages of NPs formulation, including drug loading, surface modification, or solvent exchange. Even transient exposure to these solvents can influence protein structure and protein–NPs interactions. The goal of this study is to better understand the combined effects of ACN and AuNPs on protein structure, which may offer insight into protein stability, misfolding, or aggregation mechanisms that could arise under processing or environmental stress. Furthermore, the concentration of ACN was varied from 0% to 30% vol. to investigate the effect of various solvent polarities. This approach enables us to explore how solvent-induced stress, in combination with NPs binding, affects protein folding and aggregation at various solvent polarities, a question of relevance for understanding protein-nanomaterial interactions under nonideal or stress conditions, such as in formulation environments.

Circular dichroism (CD) spectroscopy and hydrodynamic diameter distribution were used to characterize changes in the protein secondary structure and formation of the protein and AuNPs aggregates at 0–30% vol. ACN concentrations. Furthermore, MD simulations were performed to complement the results of the laboratory experiments and to investigate intermolecular interactions between AuNPs and the BSA structure in the presence of organic solvent. Considering the limitations of the MD simulation box size, one BSA monomer structure and one AuNP (d = 5 nm) were inserted in the simulation box to investigate the binding sites of the protein monomer to AuNPs at various ACN concentrations. The spectroscopy analysis conclusively showed that the presence of AuNPs caused partial unfolding of the BSA structure with increased hydrodynamic diameter distribution at various solvent polarities. Furthermore, MD simulations showed enhanced protein-ACN interactions in the presence of one AuNP at 15% vol. ACN. Overall, this combined experimental and computational study provides a comprehensive understanding of the effect of the AuNPs and coexisting organic solvent on the protein structure and associated intermolecular interactions, essential for the development of DDS with an enhanced biocompatibility and efficacy.

## 2 Methodology

### 2.1 Materials

BSA and AuNPs (diameter = 5 nm) were purchased from Sigma-Aldrich (St Louis, MO, USA). The 5 nm AuNPs used in this study were purchased as a stabilized suspension but were not citrate-capped or functionalized with other ligands. They were supplied and dispersed in low ionic strength phosphate-buffered saline (0.1 mM PBS), which provides modest colloidal stability. Sodium chloride (NaCl) was purchased from Fujifilm Wako Pure Chemical Co. (Osaka, Japan). Acetonitrile was purchased from Fujifilm Wako Pure Chemical Co. (Osaka, Japan). Phosphate buffered saline (PBS) was purchased from Otsuka Pharmaceutical Co. (Tokushima, Japan). All the reagents were used without further purification.

### 2.2 Circular Dichroism (CD) Spectroscopy

Ninety μg/mL BSA in H_2_O, 20 μg/mL AuNPs stabilized suspension in PBS, and 90 mg/mL NaCl in H_2_O were used to prepare eight types of samples by varying the presence or absence of AuNPs at various solvent concentrations, such as 0%, 5%, 15%, and 30% vol. ACN. The final concentration of BSA in the analyzed solutions was 30 μg/mL. The 5 nm AuNPs and NaCl concentrations were 2.7 μg/mL and 9 mg/mL, respectively. The samples were stirred and incubated for 48 h at 37 °C. A 3 mL sample was placed in a quartz glass cuvette with a path length of 10 mm (Q-4 Sansyo Co. Ltd., Tokyo, Japan). The measurements were performed via a circular dichroism spectrometer (J-820, JASCO Co., Tokyo, Japan). The CD spectra results were further analyzed using the BeStSel server^35^ to quantify the percentages of α-helix, β-sheet, and unstructured motifs in the BSA secondary structure.

We note that the pH of the samples used in CD spectroscopy was not actively controlled or measured, as no buffer was added to minimize spectral interference. While the pH is estimated to be in the range of 6.5–7.0, small variations cannot be excluded and may influence the protein’s net charge and its interaction with AuNPs. This is acknowledged as a limitation of the study, and future work could explore the role of controlled pH environments in greater detail.

### 2.3 Hydrodynamic Diameter Distribution

The hydrodynamic diameter distribution was measured in the solutions with albumin (0.3 mg/mL, 3.0 mg/mL) at different solvent polarities, such as ACN 0%, ACN 5%, ACN 15%, and ACN 30%, in the absence and presence of AuNPs (2.7 μg/mL). The protein and solvents were incubated at 37 °C at constant stirring for 48 h in the presence of 0.15 M NaCl. The hydrodynamic diameter distribution was recorded with polydispersity index (P.I.) by dynamic light scattering (DLS) using ELSZ-2000ZS (Otsuka Electronics Co., Ltd., Osaka, Japan). It should be noted that DLS measurements were performed once per system, and the reported hydrodynamic diameters correspond to the dominant peak with the highest intensity. As such, mean values with standard deviations from replicate measurements are not provided, which may limit statistical interpretation. Furthermore, the imaging of the samples with BSA (3.0 mg/mL) and AuNPs (2.7 μg/mL) in 0% and 15% ACN was performed after incubation at 37 °C at constant stirring for 48 h with NaCl (0.15 M). The imaging was performed by transmission electron microscopy (TEM) using H-7650 (Hitachi High-Tech, Japan).

### 2.4 MD Simulations

The protein structure of the BSA monomer was obtained from the Protein Data Bank (PDB ID: 4F5S)^36^ with a total charge of −16. The BSA structure has a high helical content and consists of 583 amino acids. The face-centered cubic structure of the AuNP was built by the simulation input generator CHARMM-GUI,^37^ and the parameters for the gold atoms were taken from the literature: δ = 0.2951 nm and ε = 21.9006 kJ/mol.^38^ To perform atomistic MD simulations, the Gromacs 2022.6 software^39^ was used with the Amber99SB force field, which was selected based on the validations from the literature.^31,40^ The simulations were performed in a 15 nm × 15 nm × 15 nm box with a randomly inserted BSA monomer and 5 nm AuNP. The equimolar concentrations of the BSA monomer and the AuNP were 0.5 mM. The mass concentration of the BSA monomer was 33 mg/mL, and the mass concentration of the AuNP was 387 mg/mL. The system was neutralized by the addition of Na^+^ ions. The MD simulations were performed in aqueous solutions containing 0.15 M NaCl to approximate the experimental ionic strength and to mimic the presence of electrolytes. While phosphate ions present in PBS were not explicitly modeled in the simulations, the key ionic strength effects were represented by NaCl. To investigate the effect of the organic solvent, the simulations were performed in the absence and presence of ACN at 5%, 15%, and 30% vol. conditions. Representative snapshots of the initial configurations of the simulated systems and the number of molecules inserted in the simulation boxes are shown in Figure S1 and Table 1.

**Table 1:** Number of Ions and Molecules Inserted in the Simulation Boxes with 0–30% Vol^a^

Solvent	BSA	Na^+^	Cl^–^	Water	ACN	AuNP
0% ACN	1	321	305	80,000	0	-/1
5% ACN	1	321	305	78,560	1440	-/1
15% ACN	1	321	305	75,360	4640	-/1
30% ACN	1	321	305	69,600	10,400	-/1

a ACN conditions.

The energy optimization step was performed, setting the maximum atomic force constraint at 100 kJ mol^–1^ nm^–1^. The constant-volume, constant-temperature (NVT) ensemble was conducted for 0.025 ns with hydrogen bonds (H-bonds) constraints at a constant temperature of 310 K, followed by a constant-pressure, constant-temperature (NPT) equilibration step performed for 0.025 ns with all-bond constraints at a constant pressure of 1 bar. For the pressure and temperature couplings, a Berendsen barostat and a V-rescale thermostat were applied, respectively,^43^ chosen based on previous literature studies.^40^ Periodic boundary conditions were applied for XYZ directions. A cutoff of 1 nm distance was used for the short-range interactions. The MD simulations were performed for 50 ns with an integration time step of 0.002 ps. The output parameters were saved every 2000 frames, corresponding to 4 ns of dynamic runs. The systems were simulated three times, starting from the random initial coordinates.

The changes in the BSA structure and possible protein unfolding in the simulated systems was characterized by time-evolution of the solvent accessible surface area (SASA) of three distinct protein domains (Domain I: with the amino-acid residues number 1–196, Domain II: a. a. 204–381, and Domain III: a. a. 382–571). In addition, the percentage change of SASA values relative to the initial SASA values at 0 ns in each corresponding system was averaged among the last 5 ns of the simulations for each MD run. The BSA binding sites were analyzed by studying the distances between the COM of the AuNP and six protein subdomains (IA: with the amino-acid residues number 1–107, IB: a. a. 108–196, IIA: a. a. 204–295, IIB: a. a. 296–381, IIIA: a. a. 382–493, IIIB: a. a. 494–571). The statistical significance of the changes in the SASA of protein domains in the simulated systems with 5 nm AuNP was confirmed using Excel’s Real Statistics Analysis Tool add-in. The normality and homogeneity of variance were confirmed using the Shapiro-Wilk and Levene’s tests, respectively. To confirm statistically significant differences, a one-way ANOVA and a posthoc Tukey HSD (Honestly Significant Difference)/Kramer test were performed, as the data were normally distributed with homogeneous variances. A statistical significance threshold of 0.05 was applied to the p-value. The Supporting Information contains the statistical analysis results.

The root-mean-square fluctuations (RMSF) analysis of the C-α amino acids of the BSA monomer was used to characterize the movement of amino acids in the simulated systems. Radial distribution function (RDF) analysis was performed to characterize the interactions between BSA subdomains and solvent molecules. Interaction energies between protein and solvent molecules were calculated in the absence and presence of AuNPs in 0–30% ACN, considering Coulomb (electrostatic) and Lennard-Jones (L-J) interaction energies. RMSF, RDF, and interaction energy analyses were averaged over the last 5 ns among three runs. A statistical analysis was performed for the interaction energies using Excel’s Real Statistics Analysis Tool add-in. Similarly, the normality and homogeneity of variance were examined by using the Shapiro-Wilk and Levene’s tests, respectively. Next, a one-way ANOVA and a posthoc Tukey HSD (honestly significant difference)/Kramer test were used to check the statistically significant difference for normally distributed data, whereas Kruskal–Wallis and a posthoc Dunn’s test were used for not normally distributed data. The average values of three different runs were reported for all of the mentioned types of analysis. Visual Molecular Dynamics (VMD) software^44^ was used to visualize the simulated systems.

## 3 Results and Discussion

### 3.1 Circular Dichroism (CD) Spectroscopy

CD spectroscopy analysis was performed to investigate the effect of 5 nm AuNPs on the changes in the secondary structure of BSA at various ACN concentrations (Figure 1). According to Figure 1A, two distinct CD spectroscopy peaks were observed at around 208 and 222 nm for the sample with BSA in 0–15% vol. ACN in the absence of AuNPs, corresponding to the α-helical structure of the protein.^27,31,45^ In particular, the quantification of the percentage composition of the protein secondary structure by the BeStSel server^35^ showed the high helical content of 59.4%, 62.6%, and 60.9% in the absence of AuNPs at 0%, 5%, and 15% ACN conditions. In comparison, in the presence of AuNPs, the helical content of protein slightly decreased to 55.5%, 57.7%, and 50.7% in 0%, 5%, and 15% ACN, respectively, indicating a partial unfolding, as was also shown by decreased ellipticity in Figure 1B–D. Moreover, an increased amounts of β-sheets (up to 31.4%) were observed in the presence of AuNPs at 15% ACN conditions, in comparison to 0% and 5% ACN conditions with the β-sheets composition of 15% and 20.5%, respectively. Furthermore, decreased ellipticity and the loss of the protein secondary structure were observed at reduced solvent polarity (30% vol. ACN), with no significant impact of AuNPs (Figure 1E). In particular, at 30% ACN, the percentage compositions of helices reduced to 42.3% and 44.4%, while the unstructured motifs increased up to 38.9% and 46.3%, in the absence and presence of AuNPs, respectively. Overall, while organic solvent induced structural changes in the albumin in 30% ACN, consistent with the previously reported changes in the secondary structure of proteins at decreased solvent polarity,^4,5^ our results suggest that the presence of AuNPs causes the loss of helical structure and partial unfolding of BSA at low ACN concentrations (in 0–15% ACN).

**Figure 1:**
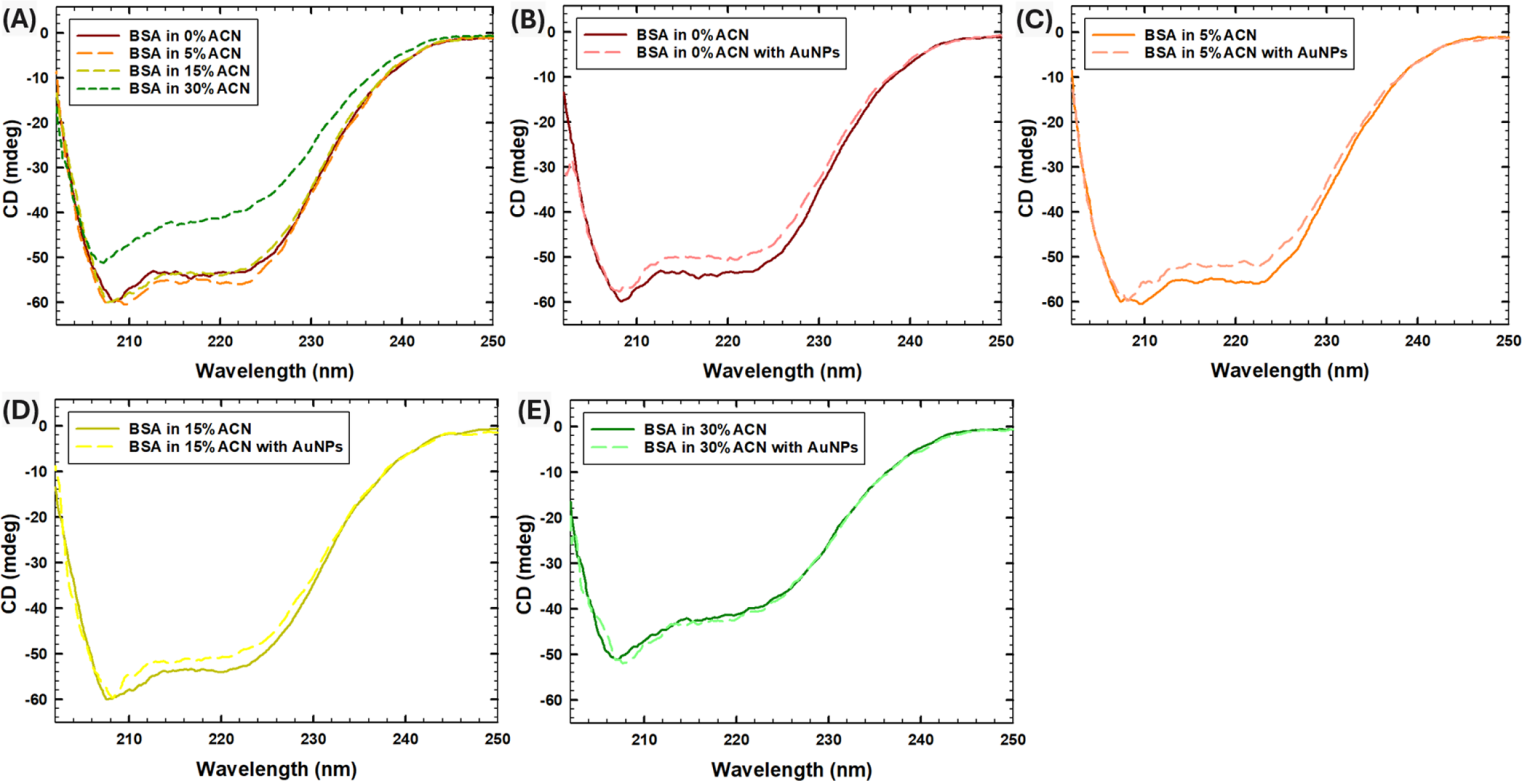
CD spectroscopy of the samples with the enlarged region around 201–260 nm: (A) in the absence of AuNPs, (B) in 0% ACN, (C) in 5% ACN, (D) in 15% ACN, (E) in 30% ACN.

### 3.2 Hydrodynamic Diameter Distribution

The hydrodynamic diameter distribution of the albumin and AuNPs aggregates was further studied at different ACN concentrations via DLS (Figure 2). According to Figure 2A,B, in the absence of AuNPs, the hydrodynamic diameter distribution of protein in 0% and 5% ACN was similar, indicated by maximum intensity values at 5.9 nm (P.I. = 0.214) and 5.7 nm (P.I. = 0.245), respectively. It should be noted that the sample with BSA in the absence of AuNPs in 0% ACN (Figure 2A) exhibited small secondary peaks in the 100–1000 nm and 1000–10,000 nm ranges, which likely correspond to a low level of protein self-aggregation. In comparison, the presence of AuNPs affected the hydrodynamic diameter distribution due to the formation of protein-NP aggregates, indicated by maximum intensity values corresponding to 10.6 nm in 0% and 5% ACN (P.I. = 0.201 and P.I. = 0.212, respectively, Figure 2E,F). In 15% ACN, in the absence of AuNPs (Figure 2C), the hydrodynamic diameter distribution of protein was around 7.4 nm (P.I. = 0.19). With the addition of AuNPs in 15% ACN, the hydrodynamic diameter distribution showed three maximum intensity peaks, corresponding to 3.7, 20.7, and 304.0 nm (P.I. = 0.19), indicating the formation of protein-NP aggregates of a larger size (Figure 2G). Considering the peak broadness observed in Figure 2E–G, the broad peaks observed in Figure 2E–F would indicate the formation of unstructured amorphous aggregates with a heterogeneous population in the presence of AuNPs at 0–5% ACN. Considering that fibrillation is a concentration-dependent process and NPs might serve as a surface for protein aggregation,^46^ more structured aggregates and ordered fibrils could be further produced from highly unfolded protein structures in the presence of AuNPs at 15% ACN, as was indicated by multiple small peaks in Figure 2G.^47^ At 30% ACN, the hydrodynamic diameter distribution was around 33.6 nm (P.I. = 0.185) and 41.0 nm (P.I. = 0.247) in the absence and presence of AuNPs, respectively (Figure 2D,H).

**Figure 2:**
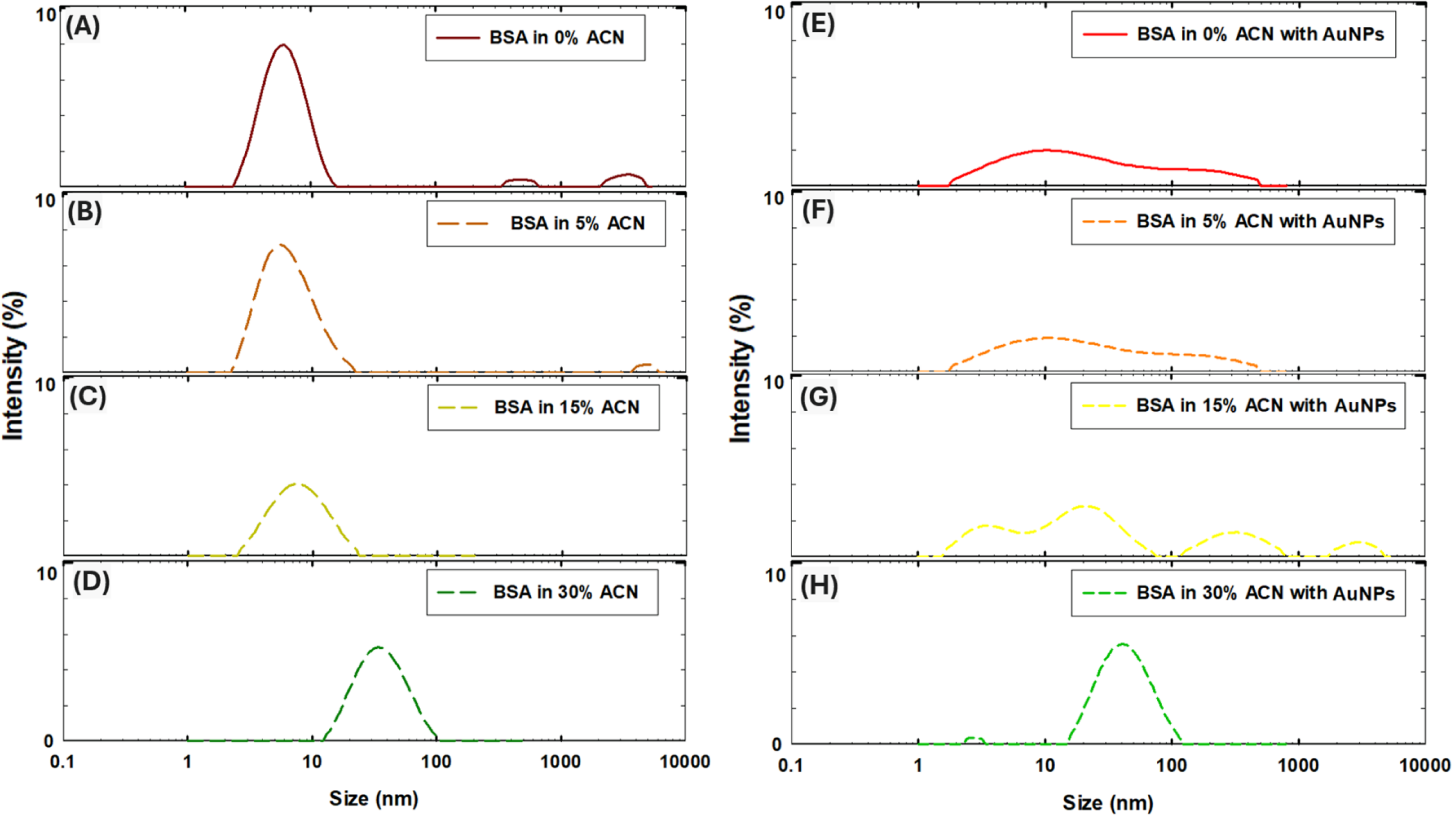
Hydrodynamic diameter distribution in 0–30% ACN: (A–D) in the absence of AuNPs, and (E–H) in the presence of AuNPs.

The results of the DLS analysis shown in Figure 2 were consistent with the previously reported findings on the enhanced formation of protein aggregates in the presence of AuNPs.^48^ Furthermore, while the presence of AuNPs increased the hydrodynamic diameter distribution due to the formation of intermolecular aggregates, the most significant effect was observed in 15% ACN, associated with the reduced solvent polarity. Imaging of the samples with BSA and AuNPs in 0% and 15% ACN was performed to provide direct visual evidence of protein-NP aggregation (Figure 3). Although such imaging data were not available for all samples in the current study due to experimental constraints, these results support and further confirm the aggregation trends observed in the DLS measurements of the corresponding samples. In particular, according to Figure 3A,B, small protein-NP clusters and few large aggregates were observed in 0% ACN, suggesting slower aggregation kinetics of proteins and NPs. In comparison, a large amount of dense amorphous network-like matrix was observed in 15% ACN, illustrating an enhanced formation of protein-NP aggregates, as shown in Figure 3C,D. Although such imaging data were not available for all samples in the current study due to experimental constraints, these results support and further confirm the aggregation trends observed in the DLS measurements of the samples with BSA and AuNPs and 0% and 15% ACN.

**Figure 3:**
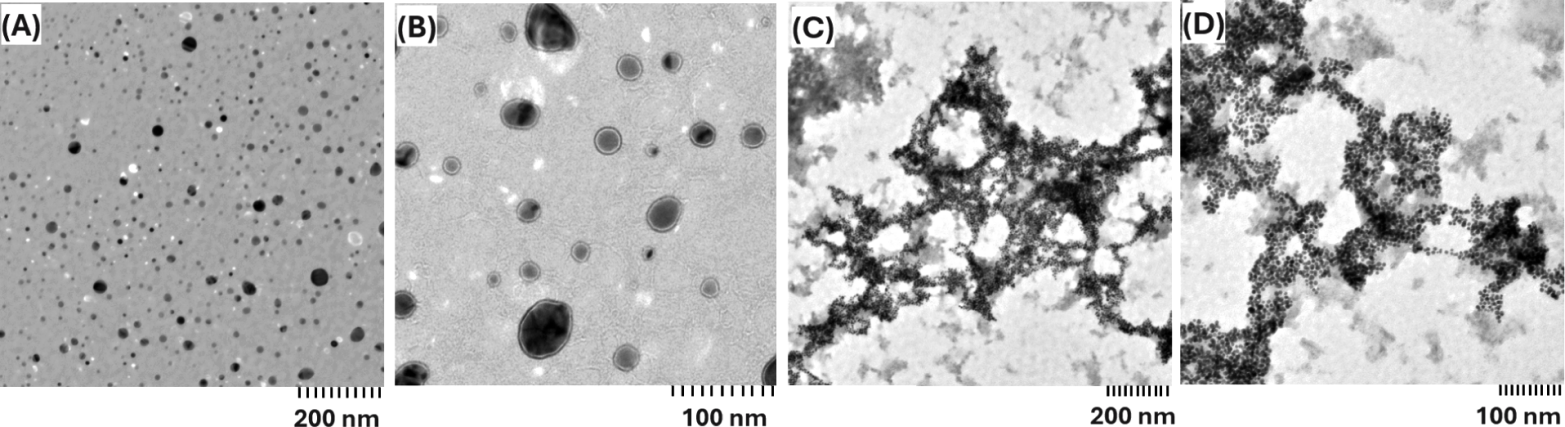
TEM images for (A,B) BSA and AuNPs in 0% ACN, (C,D) BSA with AuNPs in 15% ACN.

Such observations were consistent with the enhanced adsorption of protein on the surface of NPs at higher concentrations of organic solvent, as was previously observed by the higher protein packing density on the silica NP surface in the presence of ethanol.^49^ Moreover, in 30% ACN, the formation of protein aggregates with a comparatively larger size was observed both in the absence and presence of AuNPs, consistent with the most significant protein unfolding shown by the CD spectra analysis (Figure 1E). In this highly denaturing environment, the protein is already significantly destabilized, which may limit any further structural rearrangement upon AuNP binding. This suggests that beyond a certain threshold of organic solvent concentration, solvent effects outweigh the influence of NPs interaction, resulting in no additional conformational changes attributable to AuNPs. Nevertheless, while BSA-only controls showed aggregation at 30% ACN, indicating solvent-induced destabilization, the aggregation also occurred in the presence of both BSA and AuNPs at 0–30% ACN conditions, suggesting a synergistic effect of AuNPs and organic solvent. It should be noted, however, that AuNP-only samples in ACN were not analyzed in this study. Therefore, we cannot exclude the possibility that ACN may also affect AuNP colloidal stability. Further investigations involving NPs-only controls would help clarify the extent to which organic solvent alone contributes to aggregation behavior.

In this study, the interaction between BSA and AuNPs is governed by noncovalent physical interactions, including electrostatic attraction, van der Waals interactions, and hydrophobic contacts. Given the comparable dimensions of BSA (∼7–8 nm) and the 5 nm AuNPs, direct measurement of protein adsorption is nontrivial. In this study, interactions between BSA and AuNPs, as well as adsorption-induced conformational changes, were inferred from the observed increase in hydrodynamic diameter and solvent-dependent alterations in BSA secondary structure as determined by circular dichroism (CD) spectroscopy. However, future studies incorporating quantitative adsorption assays would further validate and expand on these findings.

### 3.3 MD Simulations

#### 3.3.1 SASA of BSA Domains, BSA Binding Sites, and Root-Mean-Square Fluctuations (RMSF) of Amino Acid Residues

To characterize the intermolecular interactions and possible unfolding of the protein monomer, three independent MD runs of 50 ns were performed with the BSA monomer structure in 0–30% ACN conditions in the presence and absence of 5 nm AuNP. The BSA protein monomer structure consists of three domains, containing six distinct protein subdomains. While the major drug-loading sites are located in subdomains IIA and IIIA, with a high propensity for binding small hydrophobic and anionic molecules, subdomain IB has been recently recognized as a binding site for endogenous ligands and complex heterocyclic drugs.^32^ To elucidate BSA binding sites to AuNPs in 0–30% ACN conditions, the intermolecular distances between the AuNP and BSA monomer subdomains were studied, considering 6 distinct subdomains of BSA (Figure 4). Furthermore, the changes in the protein solvent-accessible surface area (SASA) were investigated, considering the possible unfolding of three distinct BSA domains (Figure 5). The SASA of protein domains was averaged among three runs, and a relative percentage of SASA was reported, considering changes in the SASA values relative to the initial SASA at 0 ns in each corresponding system. Moreover, to investigate the possible correlation of unfolding of the BSA domains with the protein binding sites, the relative percentage SASA of domains was averaged among the last 5 ns for each simulation run, when the BSA and the AuNP were bound to each other (Table 2).

**Figure 4:**
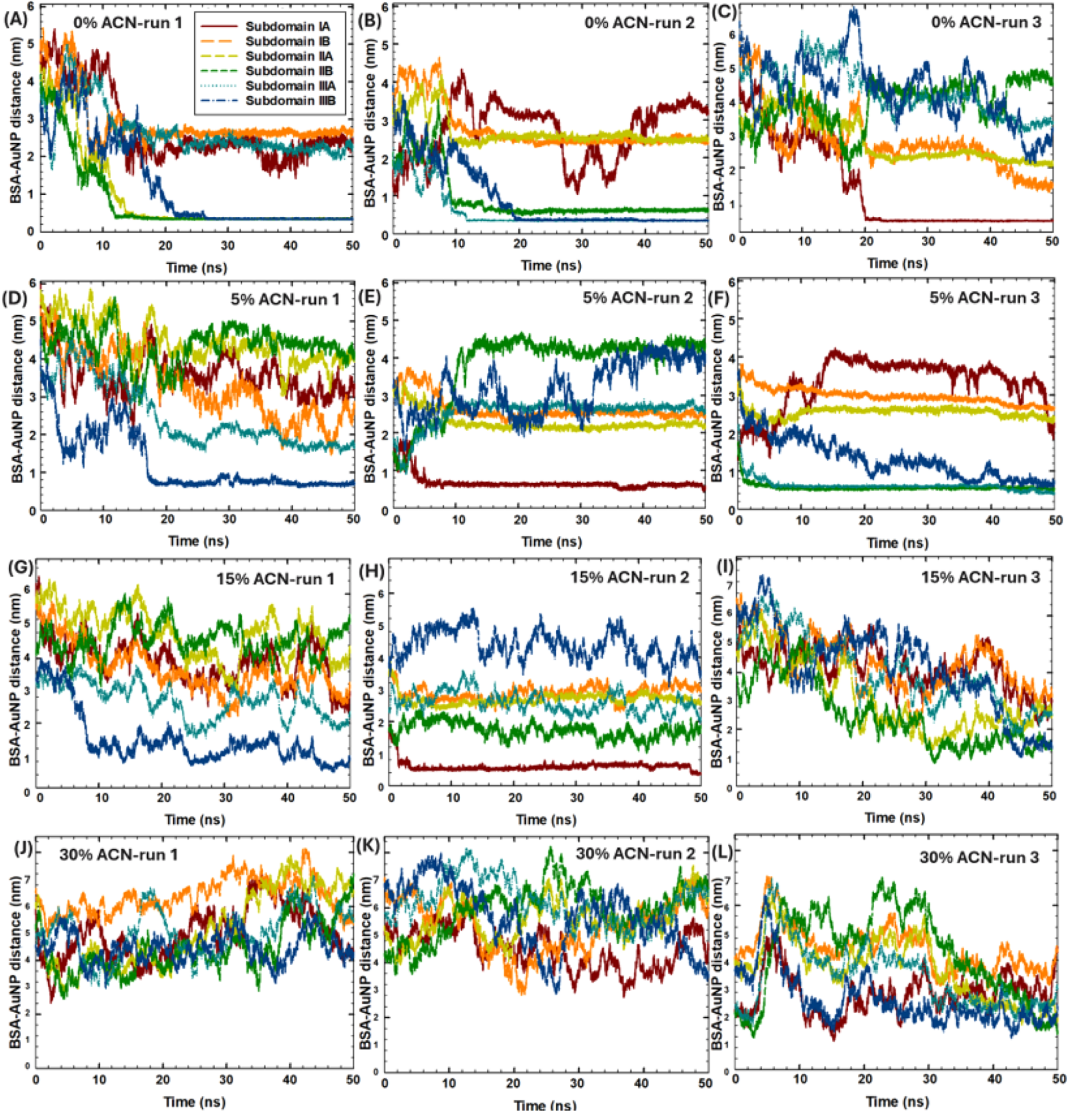
Time-evolution of the intermolecular distances between the AuNP and BSA subdomains in three MD runs: (A–C) in 0% ACN, (D–F) in 5% ACN, (G–I) in 15% ACN, (J–L) in 30% ACN (coloring methods: subdomain IA- red, IB- orange, IIA- yellow, IIB- green, IIIA- cyan, IIIB- blue).

**Figure 5:**
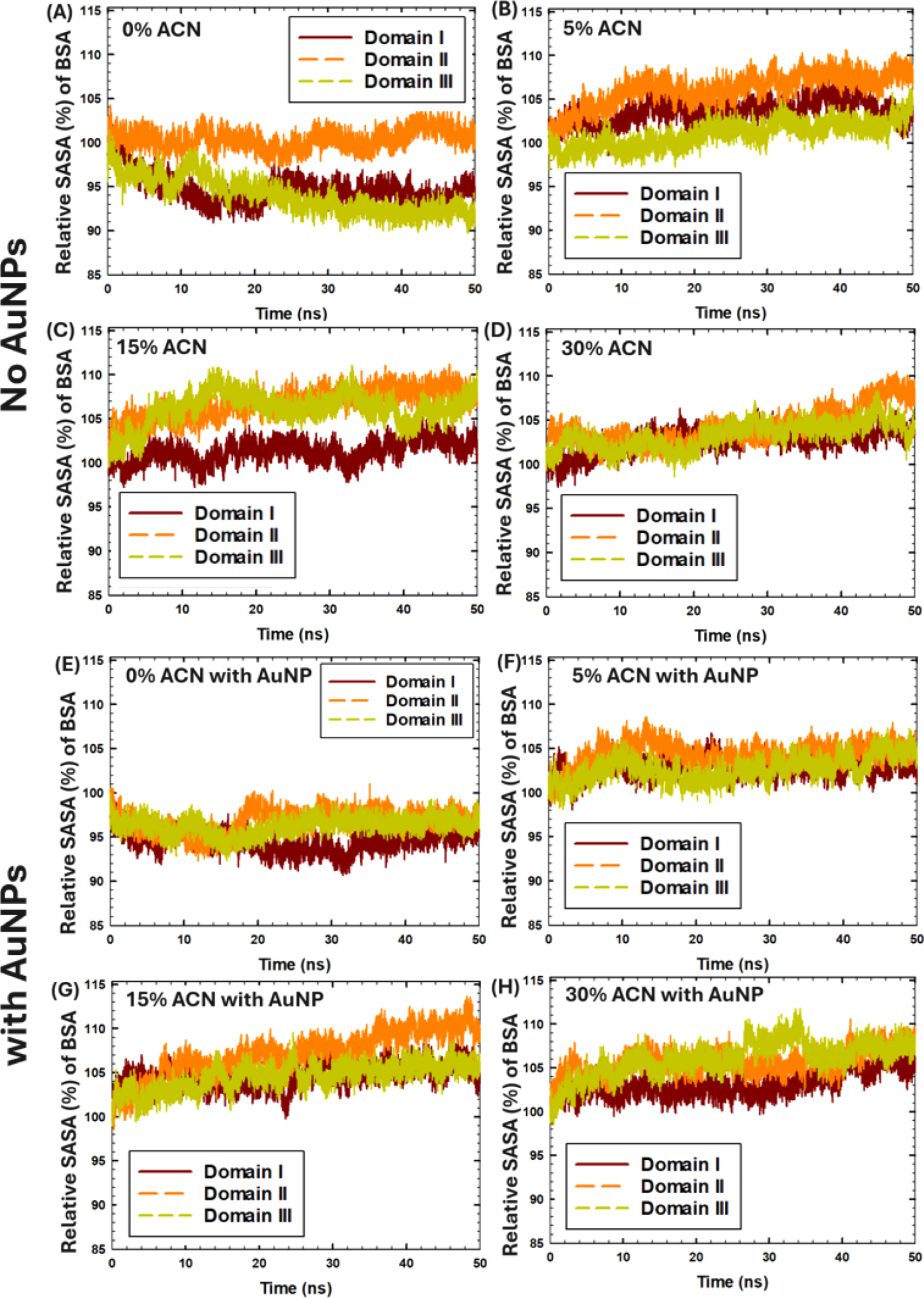
Time-evolution of the change of the SASA of the BSA domains relative to the initial SASA at 0 ns in the corresponding system, averaged among three MD runs: (A–D) in the absence of the AuNP in 0–30% ACN, (E–H) in the presence of the AuNP in 0–30% ACN.

**Table 2:** Change in the Relative Percentage of SASA of Protein Domains in the Presence ofAuNP in 0–30% ACN, Averaged among the Last 5 ns of the Simulations for Each MD Run^a^

**Domain**	**0% ACN**			**5% ACN**		
**Run**	**Run 1**	**Run 2**	**Run 3**	**Run 1**	**Run 2**	**Run 3**
**Domain I**	-1.3 ± 1.4%	-3.5 ± 1.3%	-8.6 ± 1.4%	+8.0 ± 1.5%	+0.8 ± 1.3%	+1.4 ± 1.4%
**Domain II**	+8.3 ± 1.5%	-4.2 ± 1.2%	-11.6 ± 1.1%	+5.4 ± 1.4%	+6.0 ± 1.6%	+3.2 ± 1.3%
**Domain III**	-0.5 ± 1.6%	+0.4 ± 1.4%	-9.1 ± 1.2%	+5.5 ± 1.4%	+6.9 ± 1.4%	+2.1 ± 1.4%

**Domain**	**15% ACN**			**30% ACN**		
**Run**	**Run 1**	**Run 2**	**Run 3**	**Run 1**	**Run 2**	**Run 3**
**Domain I**	+7.6 ± 1.8%	+4.4 ± 1.8%	+5.7 ± 1.6%	+2.0 ± 1.8%	+8.3 ± 1.6%	+6.7 ± 1.5%
**Domain II**	+8.5 ± 1.5%	+13.7 ± 1.7%	+9.0 ± 1.9%	+9.2 ± 1.9%	+8.5 ± 1.4%	+5.5 ± 1.2%
**Domain III**	+9.0 ± 1.4%	+2.8 ± 1.3%	+5.9 ± 1.8%	+4.5 ± 1.4%	+7.1 ± 2.0%	+10.8 ± 1.6%

a Statistically significant differences were observed in the SASA of Domain I between the samples with 15% and 30% ACN conditions in comparison to the 0% ACN environment, and in the SASA of Domain III between the simulated systems with 0% and 30% ACN.

According to Figure 4A and Table 2, in the absence of ACN (0% ACN), the AuNP was bound to subdomains IIA-B, and IIIB (in run 1), while the relative SASA of Domain-II increased by 8%, indicating the partial unfolding of Domain-II. In contrast, in run 2 (Figure 4B), when the binding sites were the subdomains IIB, and IIIA-B, the relative SASA of all domains remained low, suggesting no significant unfolding. Similarly, in run 3 (Figure 4C), when the AuNP was bound to subdomain IA, the relative SASA of all domains remained low, suggesting no significant unfolding. In the presence of organic solvent, in 5% ACN, in run 1 (Figure 4D), the AuNP was bound to subdomain IIIB, while the SASA of all domains increased by 5–8%, indicating partial unfolding from interactions with the AuNP. In run 2 (Figure 4E), when the AuNP was bound to the subdomain IA, the SASA of Domains II–III increased by 6%. In run 3 (Figure 4F), binding sites were IIB and IIIA-B, while SASA values increased only by 1–3%. In 15% ACN, in run 1 (Figure 4G), the AuNP was bound to subdomain IIIB, while the SASA of Domain III increased by 7–9%. In run 2 (Figure 4H), the SASA of Domain II increased more significantly by 13.7%, with the binding site at subdomain IA. In run 3 (Figure 4I), the binding sites were the subdomains IIB and IIIB, and the SASA of Domain II increased by 9%. In 30% ACN (Figure 4J–L), when BSA was 2–4 nm away from the AuNP, due to the thick ACN layer around the NP, no distinct binding sites were observed; and the SASA of all domains increased by 2–11%.

According to Figure 5 and Table 2, the average relative percentage SASA values of BSA domains increased in the presence of ACN and AuNPs, indicating the partial unfolding of the protein monomer. In particular, the relative SASA of Domain I increased by approximately 10% in 5–30% ACN in the absence and presence of the AuNP (Figure 5B–D and F–H, respectively). Similarly, the relative SASA of Domain II increased by 7% in the presence of 5–30% ACN, in the absence and presence of the AuNP, with the most prominent effect and an increase of 10% observed in the presence of the AuNP in 15% ACN (Figure 5G). In addition, the relative SASA of Domain III increased by 5–15% in 5–30% ACN in the absence and presence of the AuNP.

While multiple binding sites of albumin to AuNPs have been previously reported in the literature,^29,31,50^ our MD simulations showed how various solvent polarities might affect the partial unfolding of albumin domains with different binding sites. Overall, in 0% ACN and 5% ACN, the binding of the AuNP to BSA subdomains IIB and IIIA-B was associated with the comparatively low changes in the relative SASA values of all domains. In comparison, in 15% ACN, a greater unfolding of all domains was observed, independent of the BSA binding site to the AuNP, associated with the reduced solvent polarity. According to the statistical analysis performed for the systems in the presence of the AuNP in 0–30% ACN (Table S1–3), a statistically significant difference in the SASA of Domain I was observed in the presence of the AuNP in 15% and 30% ACN conditions in comparison to the 0% ACN environment. Furthermore, a statistically significant difference was observed in the SASA of Domain III in the presence of the AuNP between the simulated systems in 0% and 30% ACN. Considering the importance of protein secondary structural stability, such observation might be important for the design of PC-based DDS at various solvent polarities. The representative snapshots of the BSA-NP conjugates observed at the end of the simulations are shown in Figure 6. The movement of the protein molecule away from the AuNP surface observed in Figure 6J reflects the presence of ACN molecules at the AuNP interface at 30% ACN environment. Such observation was consistent with partial unfolding and conformational rearrangements of BSA induced by the solvent at 30% ACN conditions, as was shown by CD spectroscopy (Figure 1A,E).

**Figure 6:**
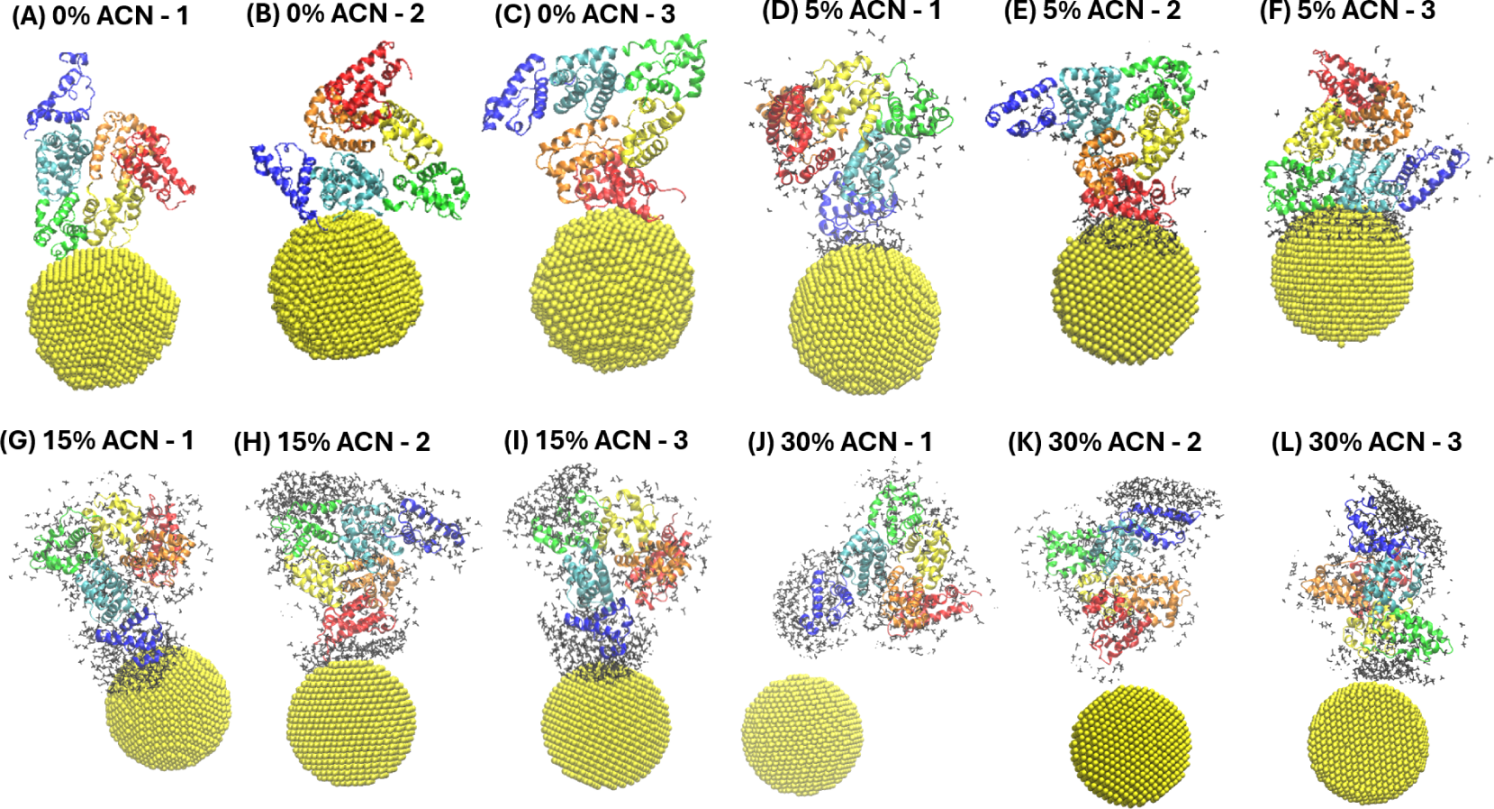
Representative snapshots of the BSA-AuNP conjugates with the ACN molecules shown within a 1 nm distance around the protein at the end of three distinct MD runs: (A–C) in 0% ACN, (D–F) in 5% ACN, (G–I) in 15% ACN, (J–L) in 30% ACN. Methods of coloring and representation style: BSA subdomains (New Cartoon representation): IA- red, IB- orange, IIA- yellow, IIB- green, IIIA- cyan, IIIB- blue; AuNP (VDW representation): yellow; ACN (licorice representation): black.

Next, the RMSF analysis was performed to investigate the effect of AuNPs on the fluctuations in the positions of BSA amino acid residues in 0–30% ACN, associated with the stability of the amino acid positions in the PC. The RMSF values were reported for the last 5 ns of the simulations, averaged among three MD runs (Figure 7). The RMSF of amino-acid residues of each MD run is shown in Figure S2. According to Figure 7A, in the absence of ACN, the presence of the AuNP increased fluctuations in Domains II–III. In particular, according to Figure S2D, the highest RMSF values were observed in run 3, when the AuNP was bound to the subdomain IA (Figure 4C). Next, while no significant changes in the RMSF values were observed with the addition of the AuNP in 5% and 30% ACN conditions (Figure 7B,D, respectively), enhanced movement of amino acids was observed in 15% ACN (Figure 7C). In particular, according to Figure S2G, the highest RMSF values were observed in runs 1 and 3, when the AuNP was bound to the subdomain IIIB (Figure 4H) and subdomain IA (Figure 4I), respectively. Similarly to 5% ACN, when the AuNP was bound to the subdomain IA in 15% ACN, the highest RMSF values were in Domains II–III, indicating the fixed position of Domain I upon binding to the AuNP. In contrast, when AuNP was bound to the subdomain IIIB in 15% ACN, the highest RMSF values were observed in all domains. Analysis of RMSF values from the MD trajectories revealed that the BSA regions in direct contact with the AuNP surface remained relatively stable throughout the simulation, with minimal fluctuation. However, enhanced flexibility was observed in regions adjacent to the binding interface, suggesting that NP binding may induce local structural rearrangements in nearby domains. This pattern supports a model in which NP interaction stabilizes the binding site while promoting conformational adaptation in neighboring flexible segments, a finding consistent with partial unfolding signatures observed in CD spectroscopy.

**Figure 7:**
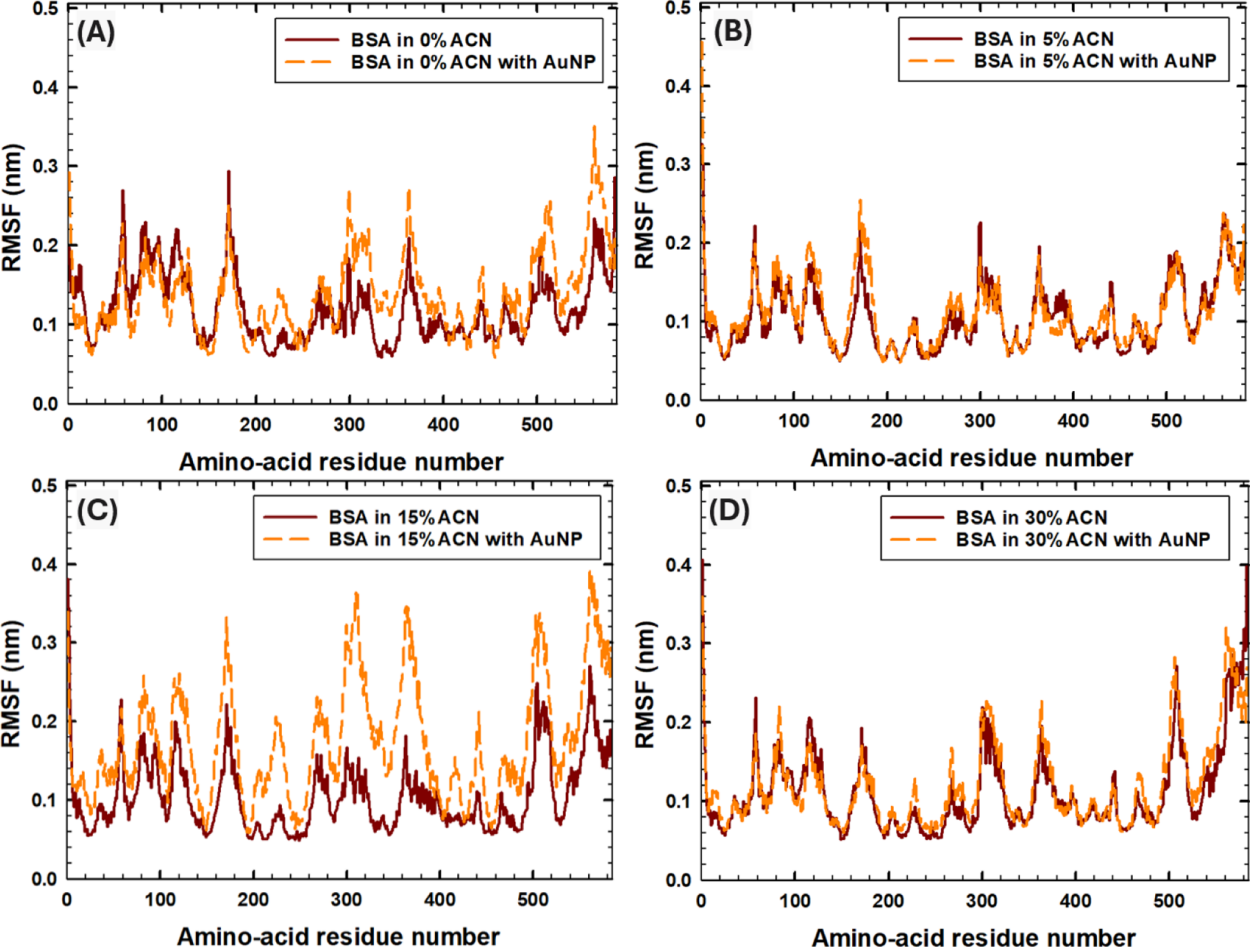
Root-mean square fluctuations of amino-acid residues in the absence and presence of AuNPs averaged over the last 5 ns among three runs: (A) in 0% ACN, (B) 5% ACN, (C)­15% ACN, and (D) 30% ACN.

#### 3.3.2 Radial Distribution Function (RDF) and Interaction Energies

The interactions between six distinct BSA subdomains and ACN in the absence and presence of AuNPs at various solvent polarities were further studied via RDF analysis, performed for the last 5 ns of the simulations, when the BSA and the AuNP were bound to each other (Figures 8–10). Furthermore, the RDF analysis was used to elucidate the effect of the presence of AuNPs on the interactions between the BSA subdomains and water at 0–30% vol. ACN in the last 5 ns of the simulations (Figures S3–6).

**Figure 8:**
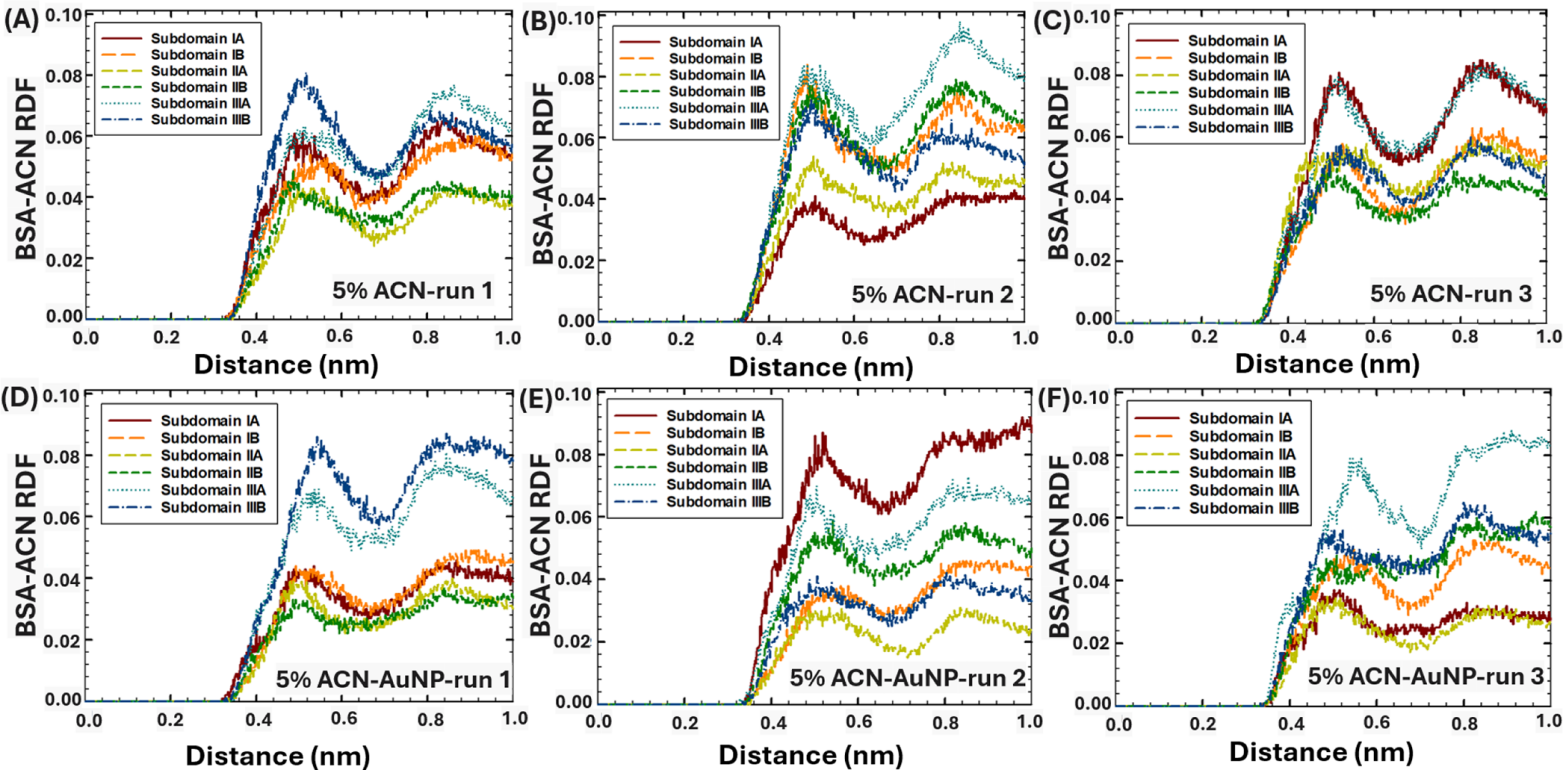
RDF of ACN and BSA subdomains in 5% ACN within 1 nm distance: (A–C) in the absence of AuNPs, and (D–F) in the presence of AuNPs.

According to the RDF analysis, in the absence of the AuNP, in 5% ACN (Figure 8A–C), ACN strongly interacted with subdomain IIIA, associated with its high hydrophobicity^51^ and propensity to attract organic solvents.^4^ In addition, in 5–30% ACN, high interactions were noted between ACN and subdomain IIIB in several runs, which were associated with its high accessibility. Moreover, in two runs in 15–30% ACN (Figures 9B and 10C), ACN strongly interacted with subdomain IIB, indicating the intercalation of ACN in protein structure at high concentrations of organic solvent. At the same time, in most runs, the weakest interactions were observed between ACN and subdomain IIA, which was associated with its lower accessibility.

**Figure 9:**
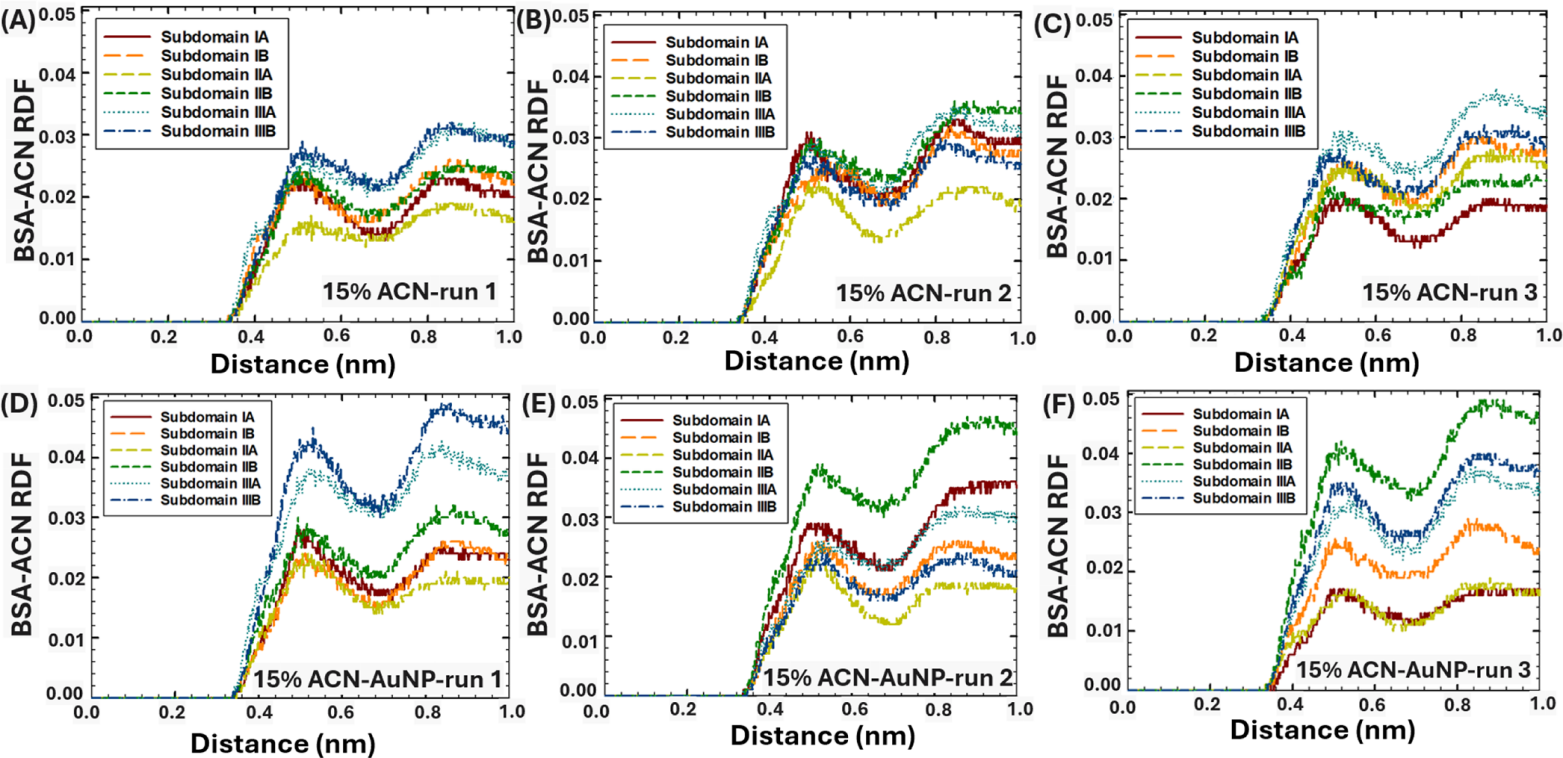
RDF of ACN and BSA subdomains in 15% ACN within 1 nm distance: (A–C) in the absence of AuNPs, and (D–F) in the presence of AuNPs.

In comparison, in the presence of the AuNP, in 5% ACN conditions (Figure 8D–F), ACN strongly interacted with subdomains IIIB (run 1), IA (run 2), and IIIA (run 3), which were established as the protein binding sites to AuNPs in each corresponding run (Figure 4D–F). This observation indicated that at low ACN concentration (5 vol %), ACN would strongly interact with the protein subdomains bound to NPs. In 15% ACN (Figure 9D–F), the interactions between ACN and BSA subdomains significantly increased, indicated by the elevated RDF peaks. In particular, the strongest interactions were observed between ACN and subdomains IIIA-B (run 1, Figure 9D) and subdomains IIB (run 2–3, Figure 9E–F). The results were in agreement with the RMSF analysis, which showed enhanced fluctuations in the positions of amino acids in the presence of the AuNP in 15% ACN (Figure 7C), which could occur due to the enhanced interactions with ACN. Interestingly, the presence of the AuNP in 30% ACN (Figure 10D–F) did not significantly affect the interaction sites and strength between ACN and BSA subdomains. This observation was consistent with the results of our experiments, where the protein structure lost its secondary structure (Figure 1E) and formed bulk aggregates (Figure 2E) both in the absence and presence of AuNPs in 30% vol. ACN conditions.

**Figure 10:**
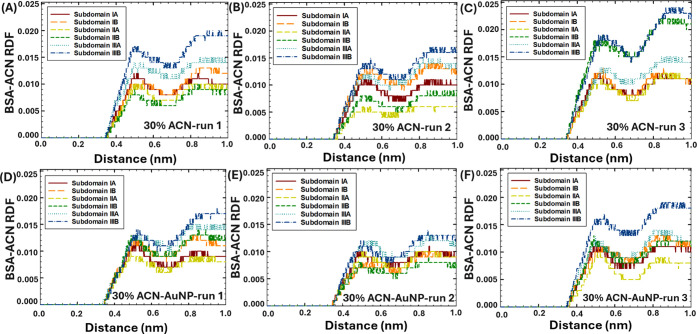
RDF of ACN and BSA subdomains in 30% ACN within 1 nm distance: (A–C) in the absence of AuNPs, and (D–F) in the presence of AuNPs.

Furthermore, although the RDF analysis showed that water molecules interacted more weakly with the subdomains IIA and IIIA in all simulated systems (Figures S3–6), the presence of AuNPs could enhance the interactions between subdomain IIIB and water when the AuNP was bound to subdomain IA in 5–15% ACN conditions (Figures S2E and S3E). Similarly, strong interactions between water molecules and subdomains IA and IIB were observed in the simulation runs when the AuNP was bound to subdomain IIIB in 5–15% ACN conditions (Figures S2D and S3D). Taking into account that while ACN would strongly interact with the protein subdomains bound to the AuNP, water molecules had a higher propensity to interact with the subdomains located slightly farther from the NP. Overall, such distinct interactions of protein subdomains with water and ACN could promote the protein partial unfolding in the presence of AuNPs at 5–15% vol. ACN concentrations.

Recent studies also showed that reduced solvent polarity suppressed the hydrophobic interactions between proteins in aqueous solutions, resulting in protein structural changes.^5^ Furthermore, small molecules could enhance the homogeneous solubility of the AuNPs in water via electrostatic repulsions, promoting nonspecific protein adsorption.^25^ In addition, strong interactions between proteins adsorbed on the silica surface were observed in the presence of ethanol at high concentrations, associated with the greater protein unfolding.^49^ Considering the different types of interactions governing the formation of PC and protein unfolding in the presence of NP and organic solvent, the effect of AuNPs on the interaction energies between the BSA monomer and solvent components was studied in 0%–30% vol. ACN. In particular, Coulomb (electrostatic) and Lennard-Jones (L-J) interaction energies were computed at the last 5 ns of the MD simulations, considering that BSA and the AuNP were bound to each other (Figure 11).

**Figure 11:**
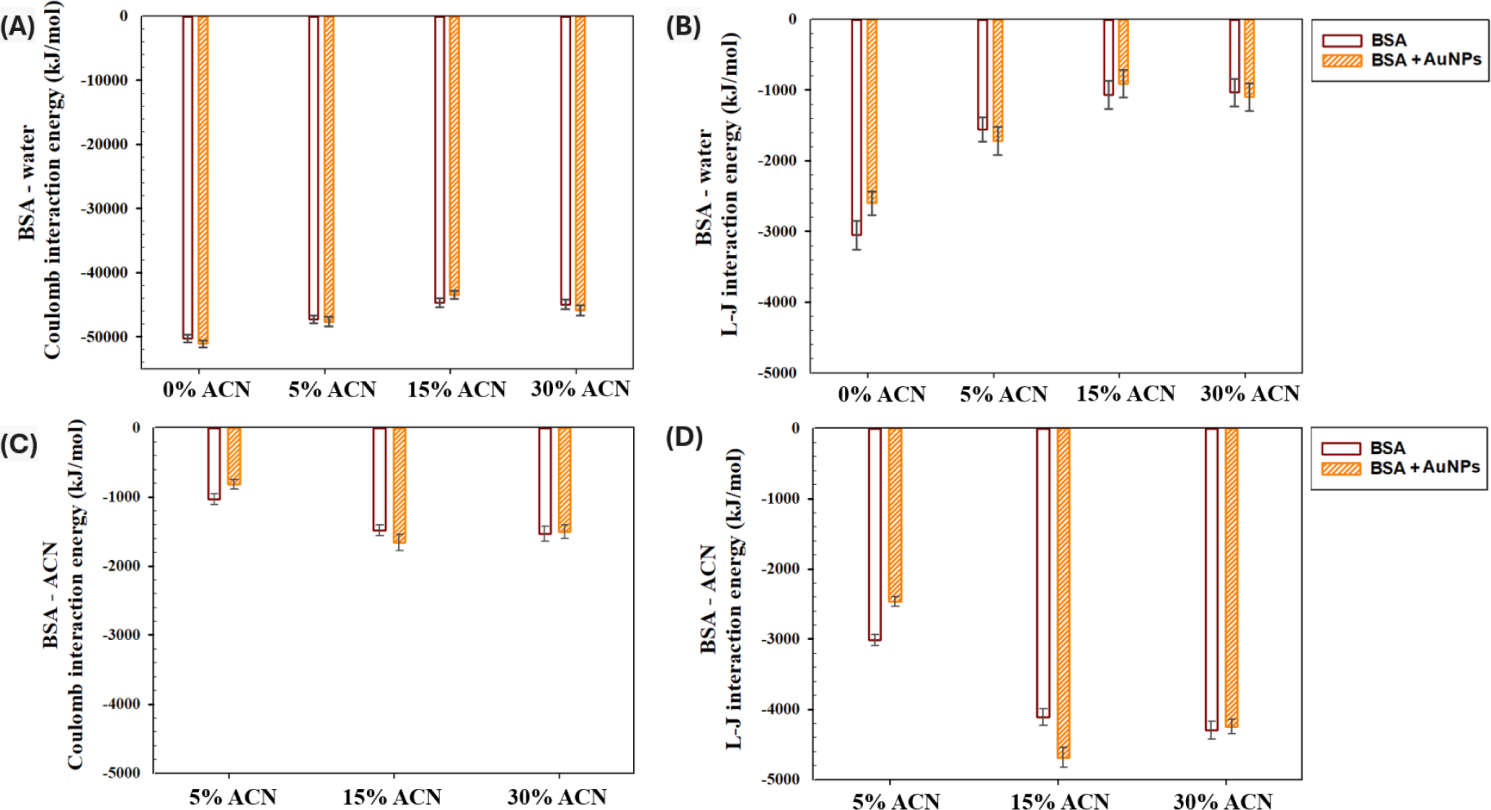
Interaction energies in the absence and presence of AuNPs in 0%–30% vol. ACN averaged over the last 5 ns among three runs: (A) BSA-water Coulomb interaction energy, (B) BSA-water Lennard-Jones interaction energy, (C) BSA-ACN Coulomb interaction energy, and (D) BSA-ACN Lennard-Jones interaction energy.

According to Figure 11, the results of our MD simulations showed an altered effect of AuNPs on the interactions between the protein monomer and solvent components. In particular, the presence of AuNPs suppressed L-J interactions between BSA and water in 0% ACN (Figure 11B), associated with the formation of the PC. In comparison, no significant effect on BSA-water interactions was observed with the addition of AuNPs in 5%–30% ACN (Figure 11A,B). Furthermore, the presence of the AuNP suppressed both Coulomb (electrostatic) and L-J interactions between BSA and ACN in 5% ACN (Figure 11C,D). In contrast, the presence of the AuNP increased the L-J interaction energy between BSA and ACN in 15% ACN, while no significant effect was observed in 30% ACN. Overall, at low ACN concentration (5% vol), the presence of the AuNP could suppress BSA-ACN and BSA-water interactions, associated with the formation of PC. In comparison, in 15% ACN, the presence of the AuNP enhanced interactions between BSA and ACN and suppressed interactions between BSA and water, consistent with the elevated RMSF values and RDF peaks at reduced solvent polarity (Figures 7C and 9). Finally, no significant effect of the AuNP on BSA-solvent interactions was observed in 30% vol. ACN, consistent with the results of the RDF analysis (Figure 10D–F), where no significant effect on the interaction sites and strength between ACN and BSA subdomains was observed in 30% ACN.

The statistical analysis showed the significant differences in the electrostatic interaction energies between BSA and water in the absence and presence of AuNPs with the addition of ACN (Tables S4–7). In addition, a statistically significant difference in the L-J interaction energies between BSA and water was observed with the addition of 15–30% ACN. Furthermore, a statistically significant difference in the L-J interaction energies between BSA and ACN was observed in the absence of the AuNPs with the increase of ACN concentration from 5% to 15–30% vol. In comparison, in the presence of the AuNP, both Coulomb and L-J interactions were statistically significantly affected by the increase of ACN concentration from 5% to 15–30% vol. Overall, while the change in the ACN concentration could alter the interaction strength between BSA and solvent components, the presence of the AuNP could also significantly enhance the electrostatic interactions between BSA and ACN in 15% ACN, associated with protein unfolding and formation of large intermolecular aggregates. Due to computational limitations, only a single BSA molecule was simulated in interaction with the 5 nm AuNP. Future simulations incorporating multiple proteins will be useful for studying cooperative or competitive adsorption effects and protein–protein interactions on the nanoparticle surface.

In the present study, MD simulations were performed in aqueous solutions containing 0.15 M NaCl, matching the physiological ionic strength set in the experiments. As reported in previous studies showed phosphate ions can also influence protein structure and interactions with NPs,^41,42^ phosphate and other buffer components may additionally affect protein structure and interactions with AuNPs. In addition, although in this study we used ACN as an organic solvent, the use of other organic solvents, such as acetone, ethanol, or ethyl acetate, might influence the outcomes of similar experiments. In particular, the physicochemical properties of the organic solvents, such as polarity, dielectric constant, and hydrogen bonding capacity, can significantly alter protein conformation and protein-NPs interactions.^5^ For example, the reduced solvent polarity might affect the solvation environment, and the local microenvironment around Trp-residues, and alter the protein conformation by protein partial unfolding.^4,52^ Ethyl acetate, being less polar than ACN, ethanol, and acetone, may lead to different degrees of protein denaturation or aggregation,^5^ thereby altering protein-NPs interactions. Furthermore, the hydrogen bonding capacity of solvents might influence the affinity between solvents and proteins, altering protein stability.^53^ In addition, protic and aprotic solvents might also alter the protein conformational dynamics and unfolding.^54^ While a selection of an optimal solvent in DDS is not yet standardized, particularly in the context of protein-NP systems, a systematic approach for selecting an optimal organic solvent may involve the consideration of the synergistic biocompatibility of NPs and coexisting solvents, especially for biological or environmental applications. A systematic comparative study involving a range of organic cosolvents would provide valuable insight into the solvent-specific mechanisms governing protein structural changes and nanoparticle surface dynamics. We recommend such investigations as an important future direction to broaden the understanding of solvent effects in protein–NPs systems.

## 4 Conclusions

In summary, the results of the CD spectroscopy conclusively showed that the presence of AuNPs caused partial unfolding of the BSA structure with increased hydrodynamic diameter distribution in the presence of 0–15% vol. ACN. Furthermore, our MD simulations revealed that while ACN would strongly interact with the BSA subdomains bound to the AuNP, water molecules had a higher propensity to interact with the subdomains located slightly farther from the NP. In general, such distinct interactions of protein monomer with water and organic solvent molecules could promote the partial unfolding of the protein in the presence of AuNPs at 5–15% vol. ACN environment. Moreover, the most prominent effect was observed at 15% vol. ACN, which was associated with the enhanced protein-ACN interactions in the presence of the AuNP. Consequently, while organic solvents might induce structural changes in the proteins at reduced solvent polarity, the presence of NPs may alter the protein–solvent interactions, enhancing protein unfolding and formation of large intermolecular aggregates. Overall, this combined experimental and computational study provides comprehensive insights into the interaction modes among protein, NP, and organic solvent, associated with the protein structural changes, essential for the development of DDS with an enhanced biocompatibility and efficacy.

## Supplementary Material



## Notes

Figure S1: representative snapshots of the simulated systems at the beginning of the MD runs; Figure S2: RMSF analysis of amino acid residues in 0–30% ACN; Figure S3–S6: RDF between BSA subdomains and water in 0–30% ACN; Tables S1–S3: statistical analyses of the significant differences in the SASA of protein domains in the presence of AuNPs; Tables S4–S7: statistical analyses of the interaction energies between protein and solvent components (PDF)
