# Supplementary material for: Effect of Gold Nanoparticles and Coexisting Acetonitrile Solvent on the Structure of Bovine Serum Albumin
Source: ACS Omega. 2025 Oct 10;10(41):48370–84. doi: 10.1021/acsomega.5c05552 (PMC12547562; doi:10.1021/acsomega.5c05552)
Supplement: Supplementary file 1 [file ao5c05552_si_001.pdf]

Effect of gold nanoparticles and co-existing acetonitrile solvent on the structure of bovine serum albumin.

Supporting Information

Samal Kaumbekova[1,2], Kyoko Omata[3], Ryo Nagasawa[3], and Masakazu Umezawa[1,3*]

[1]Department of Medical and Robotic Engineering Design, Faculty of Advanced Engineering, Tokyo University of Science, 6-3-1 Niijuku, Katsushika, Tokyo 125-8585, Japan.

[2]Department of Chemistry, Faculty of Natural Sciences, L. N. Gumilyov Eurasian National University, Astana 010000, Kazakhstan.

[3]Department of Materials Science and Technology, Graduate School of Advanced Engineering, Tokyo University of Science, 6-3-1 Niijuku, Katsushika, Tokyo 125-8585, Japan.

Key Words: gold nanoparticle; desolvation; acetonitrile; organic solvent; serum albumin; protein secondary structure; spectroscopic analysis; molecular dynamics simulations, protein corona

*Corresponding author: Masakazu Umezawa, Tokyo University of Science, 6-3-1 Niijuku, Katsushika, Tokyo 125-8585, Japan.

E-mail: masa-ume@rs.tus.ac.jp

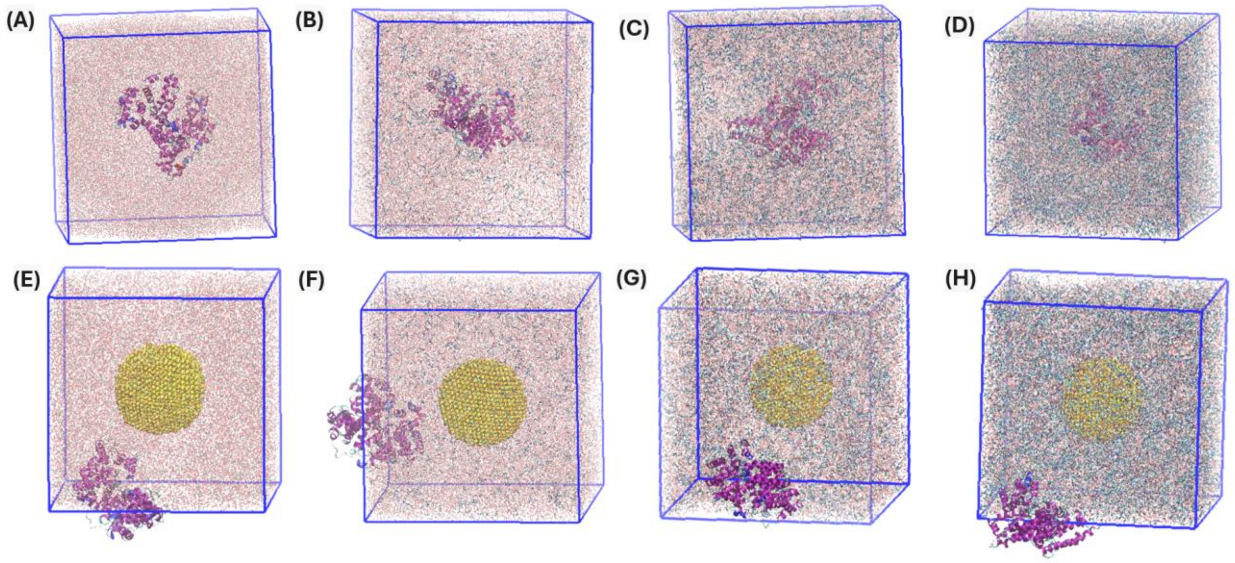

Figure S1. Representative snapshots of the simulated systems at the beginning of the MD runs in A-D) in the absence of AuNP and E-H) in the presence of AuNP; (A and E) 0% ACN, (B and F) 5% ACN, (C and G) 15% ACN, (D and H) 30% ACN. <u>Methods of coloring and representation style:</u> BSA protein (New Cartoon representation): helices = purple, red, and blue, unstructured bend and turn = cyan and white; AuNP = yellow (VDW representation); ACN = blue (CPK representation); water = red (transparent CPK representation)

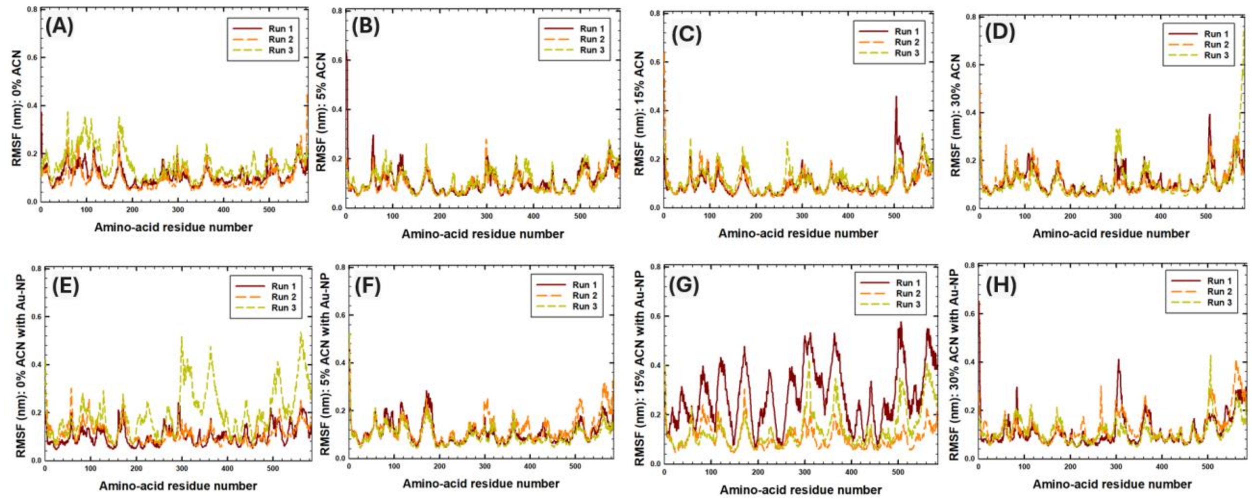

Figure S2. Root-mean square fluctuations of amino-acid residues averaged over the last 5 ns for each individual run in 0-30% ACN: A-D) in the absence of AuNP and E-H) in the presence of Au-NP

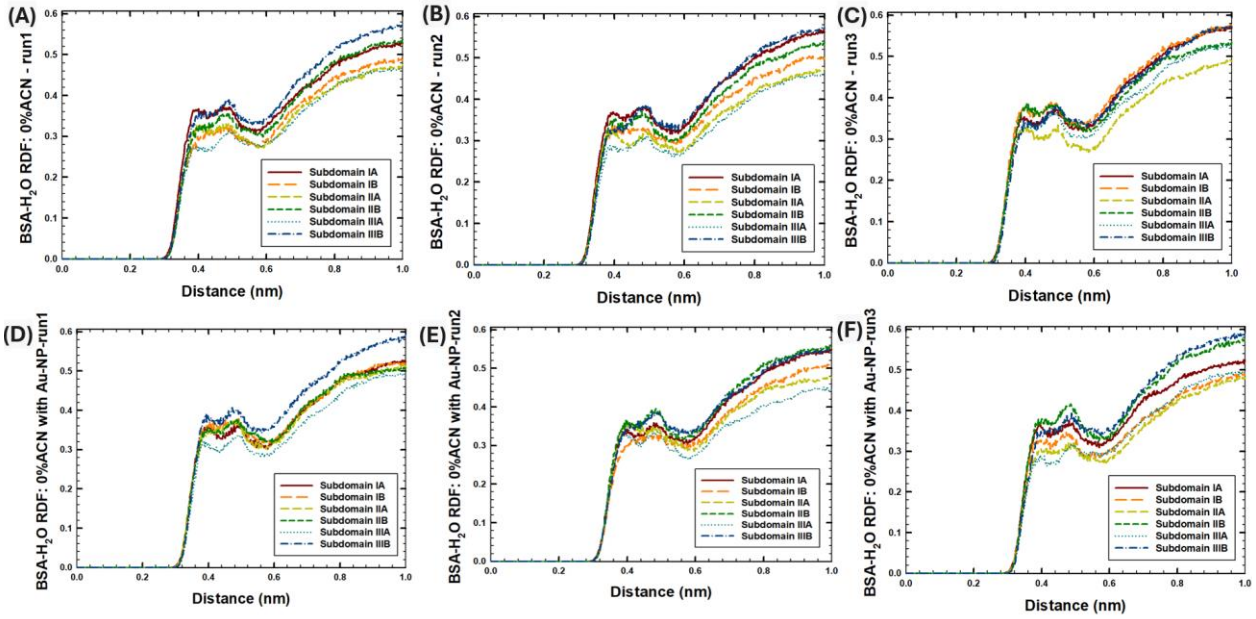

Figure S3. RDF between BSA subdomains and water in 0% ACN: A-C) in the absence of AuNP, runs 1-3, D-F) in the presence of AuNP, runs 1-3

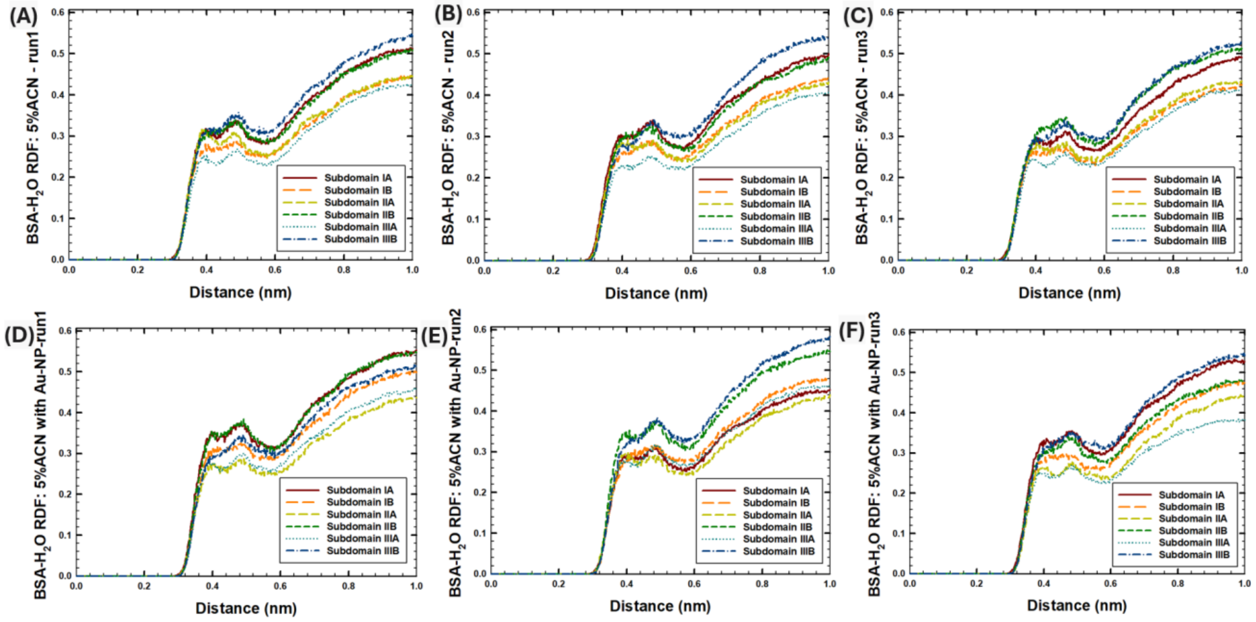

Figure S4. RDF between BSA subdomains and water in 5% ACN : A-C) in the absence of AuNP, runs 1-3, D-F) in the presence of AuNP, runs 1-3

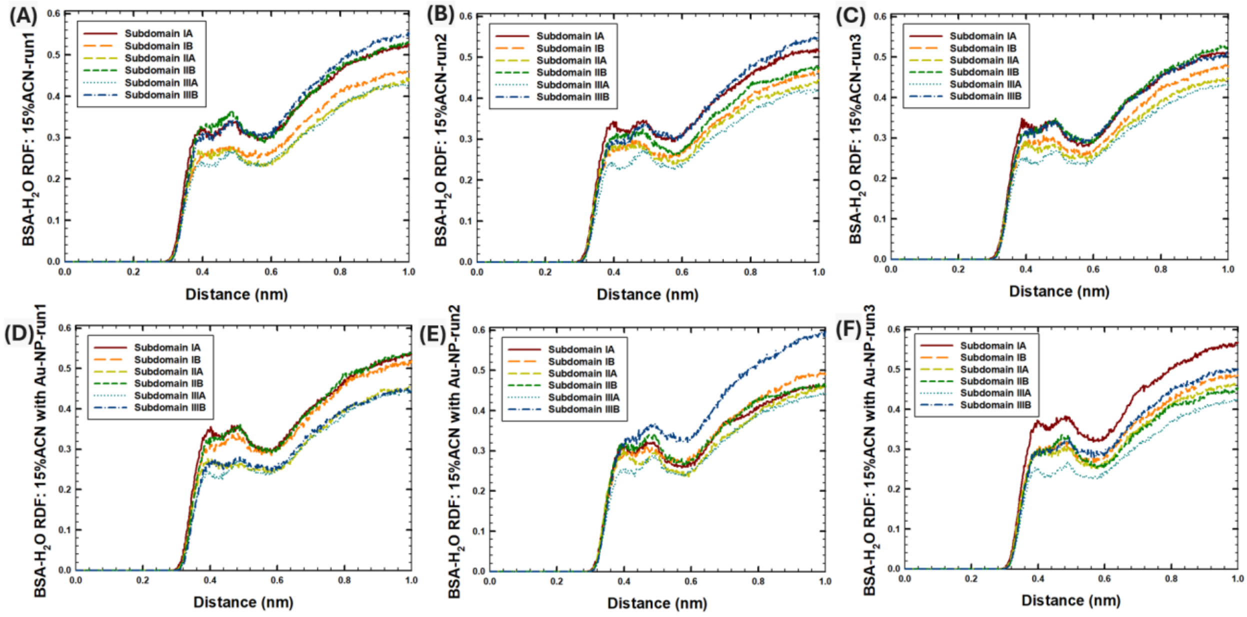

Figure S5. RDF between BSA subdomains and water in 15% ACN: A-C) in the absence of AuNP, runs 1-3, D-F) in the presence of AuNP, runs 1-3

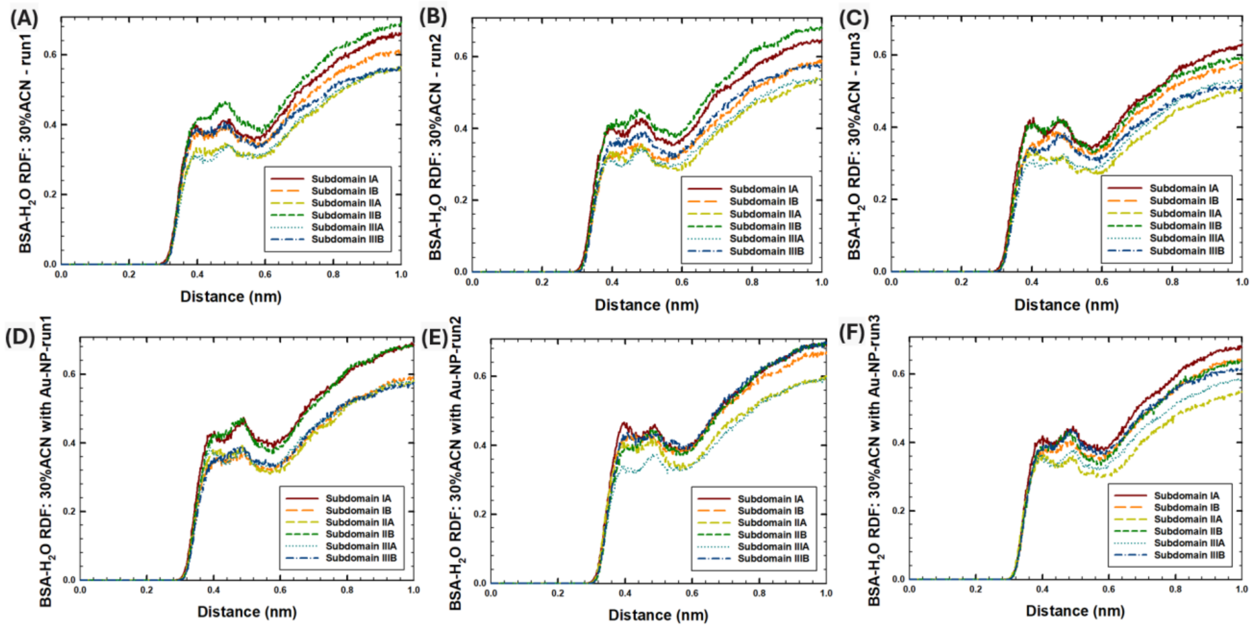

Figure S6. RDF between BSA subdomains and water in 30% ACN: A-C) in the absence of AuNP, runs 1-3, D-F) in the presence of AuNP, runs 1-3

Statistical Analyses

Table S1. Solvent Accessible Surface Area of Domains I at 0%-30% ACN

Domain I	0% ACN	5% ACN	15% ACN	30% ACN
Run 1	98.7	108	107.6	102
Run 2	96.5	100.8	104.4	108.3
Run 3	91.4	101.4	105.7	106.7

Descriptive Statistics

	0% ACN	*5% ACN*	*15% ACN*	*30% ACN*
Mean	95.53333	103.4	105.9	105.6667
Standard Error	2.162046	2.306513	0.929157	1.89062
Median	96.5	101.4	105.7	106.7
Mode	#N/A	#N/A	#N/A	#N/A
Standard Deviation	3.744774	3.994997	1.609348	3.27465
Sample Variance	14.02333	15.96	2.59	10.72333
Kurtosis	#DIV/0!	#DIV/0!	#DIV/0!	#DIV/0!
Skewness	-1.08421	1.688202	0.550596	-1.2786
Range	7.3	7.2	3.2	6.3
Maximum	98.7	108	107.6	108.3
Minimum	91.4	100.8	104.4	102
Sum	286.6	310.2	317.7	317
Count	3	3	3	3
Geometric Mean	95.48392	103.3493	105.8919	105.6325
Harmonic Mean	95.43404	103.2993	105.8837	105.5981
AAD	2.755556	3.066667	1.133333	2.444444
MAD	2.2	0.6	1.3	1.6
IQR	3.65	3.6	1.6	3.15

Shapiro-Wilk Test

	0% ACN	*5% ACN*	*15% ACN*	*30% ACN*
W-stat	0.950024	0.81203	0.988417	0.925319
p-value	0.569404	0.143553	0.794053	0.47135
alpha	0.05	0.05	0.05	0.05
normal	yes	yes	yes	yes

Levene's Tests

type	p-value
means	0.353028
medians	0.903396
trimmed	0.353028

Bartlett

0.726454

p-value >0.05
homogeneous variance

ANOVA: Single Factor

DESCRIPTION					Alpha	0.05		
Group	*Count*	*Sum*	*Mean*	*Variance*	*SS*	*Std Err*	*Lower*	*Upper*
0% ACN	3	286.6	95.53333	14.02333	28.04667	1.899488	91.15311	99.91356
5% ACN	3	310.2	103.4	15.96	31.92	1.899488	99.01977	107.7802
15% ACN	3	317.7	105.9	2.59	5.18	1.899488	101.5198	110.2802
30% ACN	3	317	105.6667	10.72333	21.44667	1.899488	101.2864	110.0469

ANOVA

Sources	*SS*	*df*	*MS*	*F*	*P value*	*Eta-sq*	*RMSSE*	*Omega Sq*
Between Groups	212.6092	3	70.86972	6.547361	0.015128	0.710586	1.477313	0.581036
Within Groups	86.59333	8	10.82417					
Total	299.2025	11	27.20023					

TUKEY HSD/KRAMER

		alpha	0.05		
group	*mean*	*n*	*ss*	*df*	*q-crit*
0% ACN	95.53333	3	28.04667		
5% ACN	103.4	3	31.92		
15% ACN	105.9	3	5.18		
30% ACN	105.6667	3	21.44667		
		12	86.59333	8	4.529

Q TEST

group 1	*group 2*	*mean*	*std err*	*q-stat*	*lower*	*upper*	*p-value*	*mean-crit*
0% ACN	5% ACN	7.866667	1.899488	4.141466	-0.73612	16.46945	0.073589	8.602782
0% ACN	15% ACN	10.36667	1.899488	5.45761	1.763884	18.96945	0.020138	8.602782
0% ACN	30% ACN	10.13333	1.899488	5.33477	1.530551	18.73612	0.022664	8.602782
5% ACN	15% ACN	2.5	1.899488	1.316144	-6.10278	11.10278	0.789976	8.602782
5% ACN	30% ACN	2.266667	1.899488	1.193304	-6.33612	10.86945	0.832565	8.602782
15% ACN	30% ACN	0.233333	1.899488	0.12284	-8.36945	8.836116	0.999744	8.602782

Table S2. Solvent Accessible Surface Area of Domains II at 0%-30% ACN

Domain II	0% ACN	5% ACN	15% ACN	30% ACN
Run 1	108.3	105.4	108.5	109.2
Run 2	95.8	106	113.7	108.5
Run 3	88.4	103.2	109	105.5

Descriptive Statistics

	0% ACN	*5% ACN*	*15% ACN*	*30% ACN*
Mean	97.5	104.8667	110.4	107.7333
Standard Error	5.807179	0.851143	1.656301	1.134803
Median	95.8	105.4	109	108.5
Mode	#N/A	#N/A	#N/A	#N/A
Standard Deviation	10.05833	1.474223	2.868798	1.965536
Sample Variance	101.17	2.173333	8.23	3.863333
Kurtosis	#DIV/0!	#DIV/0!	#DIV/0!	#DIV/0!
Skewness	0.738838	-1.41491	1.673048	-1.4882
Range	19.9	2.8	5.2	3.7
Maximum	108.3	106	113.7	109.2
Minimum	88.4	103.2	108.5	105.5
Sum	292.5	314.6	331.2	323.2
Count	3	3	3	3
Geometric Mean	97.15879	104.8597	110.3754	107.7213
Harmonic Mean	96.82341	104.8528	110.351	107.7092
AAD	7.2	1.111111	2.2	1.488889
MAD	7.4	0.6	0.5	0.7
IQR	9.95	1.4	2.6	1.85

Shapiro-Wilk Test

	0% ACN	*5% ACN*	*15% ACN*	*30% ACN*
W-stat	0.978576	0.90184	0.821385	0.885893
p-value	0.719444	0.391383	0.166644	0.341908
alpha	0.05	0.05	0.05	0.05
normal	yes	yes	yes	yes

Levene's Tests

type	p-value
means	0.057369
medians	0.236877
trimmed	0.057369

Bartlett

0.054814

p-value >0.05
homogeneous variance

ANOVA: Single Factor

DESCRIPTION Alpha 0.05

Group	Count	Sum	Mean	Variance	SS	Std Err	Lower	Upper
0% ACN	3	292.5	97.5	101.17	202.34	3.101568	90.34777	104.6522
5% ACN	3	314.6	104.8667	2.173333	4.346667	3.101568	97.71444	112.0189
15% ACN	3	331.2	110.4	8.23	16.46	3.101568	103.2478	117.5522
30% ACN	3	323.2	107.7333	3.863333	7.726667	3.101568	100.5811	114.8856

ANOVA

Sources	SS	df	MS	F	P value	Eta-sq	RMSSE	Omega Sq
Between Groups	278.5092	3	92.83639	3.216877	0.082781	0.546758	1.035515	0.35659
Within Groups	230.8733	8	28.85917					
Total	509.3825	11	46.3075					

TUKEY HSD/KRAMER

alpha 0.05

group	mean	n	ss	df	q-crit
0% ACN	97.5	3	202.34		
5% ACN	104.8667	3	4.346667		
15% ACN	110.4	3	16.46		
30% ACN	107.7333	3	7.726667		
		12	230.8733	8	4.529

Q TEST

group 1	group 2	mean	std err	q-stat	lower	upper	p-value	mean-crit
0% ACN	5% ACN	7.366667	3.101568	2.375143	-6.68033	21.41367	0.392382	14.047
0% ACN	15% ACN	12.9	3.101568	4.159187	-1.147	26.947	0.072298	14.047
0% ACN	30% ACN	10.23333	3.101568	3.299407	-3.81367	24.28033	0.16928	14.047
5% ACN	15% ACN	5.533333	3.101568	1.784044	-8.51367	19.58033	0.609184	14.047
5% ACN	30% ACN	2.866667	3.101568	0.924264	-11.1803	16.91367	0.911395	14.047
15% ACN	30% ACN	2.666667	3.101568	0.85978	-11.3803	16.71367	0.92671	14.047

Table S3. Solvent Accessible Surface Area of Domains II at 0%-30% ACN

Domain III	0% ACN	5% ACN	15% ACN	30% ACN
Run 1	99.5	105.5	109	104.5
Run 2	100.4	106.9	102.8	107.1
Run 3	105.7	102.1	105.9	110.8

Descriptive Statistics

	0% ACN	*5% ACN*	*15% ACN*	*30% ACN*
Mean	101.8667	104.8333	105.9	107.4667
Standard Error	1.934195	1.425171	1.789786	1.827871
Median	100.4	105.5	105.9	107.1
Mode	#N/A	#N/A	#N/A	#N/A
Standard Deviation	3.350124	2.468468	3.1	3.165965
Sample Variance	11.22333	6.093333	9.61	10.02333
Kurtosis	#DIV/0!	#DIV/0!	#DIV/0!	#DIV/0!
Skewness	1.592483	-1.12668	-6.2E-14	0.514178
Range	6.2	4.8	6.2	6.3
Maximum	105.7	106.9	109	110.8
Minimum	99.5	102.1	102.8	104.5
Sum	305.6	314.5	317.7	322.4
Count	3	3	3	3
Geometric Mean	101.8304	104.8138	105.8697	107.4357
Harmonic Mean	101.7945	104.7942	105.8395	107.4048
AAD	2.555556	1.822222	2.066667	2.222222
MAD	0.9	1.4	3.1	2.6
IQR	3.1	2.4	3.1	3.15

Shapiro-Wilk Test

	0% ACN	*5% ACN*	*15% ACN*	*30% ACN*
W-stat	0.856252	0.945295	1	0.98994
p-value	0.257316	0.549125	0.999999	0.80812
alpha	0.05	0.05	0.05	0.05
normal	yes	yes	yes	yes

Levene's Tests

type	p-value
means	0.936144
medians	0.989062
trimmed	0.936144

Bartlett

0.982911

p-value >0.05
homogeneous variance

ANOVA: Single Factor

DESCRIPTION					Alpha	0.05		
Group	*Count*	*Sum*	*Mean*	*Variance*	*SS*	*Std Err*	*Lower*	*Upper*
0% ACN	3	290.8	96.93333	27.50333	55.00667	2.106142	92.07656	101.7901
5% ACN	3	314.5	104.8333	6.093333	12.18667	2.106142	99.97656	109.6901
15% ACN	3	317.7	105.9	9.61	19.22	2.106142	101.0432	110.7568
30% ACN	3	322.4	107.4667	10.02333	20.04667	2.106142	102.6099	112.3234

ANOVA

Sources	*SS*	*df*	*MS*	*F*	*P value*	*Eta-sq*	*RMSSE*	*Omega Sq*
Between Groups	198.2167	3	66.07222	4.965036	0.031115	0.65058	1.286473	0.497805
Within Groups	106.46	8	13.3075					
Total	304.6767	11	27.69788					

TUKEY HSD/KRAMER

		alpha	0.05		
group	*mean*	*n*	*ss*	*df*	*q-crit*
0% ACN	96.93333	3	55.00667		
5% ACN	104.8333	3	12.18667		
15% ACN	105.9	3	19.22		
30% ACN	107.4667	3	20.04667		
		12	106.46	8	4.529

Q TEST

group 1	*group 2*	*mean*	*std err*	*q-stat*	*lower*	*upper*	*p-value*	*mean-crit*
0% ACN	5% ACN	7.9	2.106142	3.750935	-1.63872	17.43872	0.108627	9.538716
0% ACN	15% ACN	8.966667	2.106142	4.25739	-0.57205	18.50538	0.065541	9.538716
0% ACN	30% ACN	10.53333	2.106142	5.001246	0.994617	20.07205	0.031349	9.538716
5% ACN	15% ACN	1.066667	2.106142	0.506455	-8.47205	10.60538	0.983147	9.538716
5% ACN	30% ACN	2.633333	2.106142	1.250312	-6.90538	12.17205	0.813222	9.538716
15% ACN	30% ACN	1.566667	2.106142	0.743856	-7.97205	11.10538	0.95037	9.538716

Table S4. Coulombic interactions between BSA and water

BSA-water	In the absence of Au-NP				In the presence of Au-NP			
Coulomb	0% ACN no AuNP	5% ACN no AuNP	15% ACN no AuNP	30% ACN no AuNP	0% ACN with AuNP	5% ACN with AuNP	15% ACN with AuNP	30% ACN with AuNP
Run 1	-48995	-48088	-45014	-45690	-51475	-48255	-43560	-45359
Run 2	-50351	-46575	-45437	-44368	-51636	-47840	-43210	-46616
Run 3	-51534	-47248	-43702	-44895	-50231	-46829	-43484	-45697

Descriptive Statistics

	0% ACN no AuNP	*5% ACN no AuNP*	*15% ACN no AuNP*	*30% ACN no AuNP*	*0% ACN with AuNP*	*5% ACN with AuNP*	*15% ACN with AuNP*	*30% ACN with AuNP*
Mean	-50293.3	-47303.7	-44717.7	-44984.3	-51114	-47641.3	-43418	-45890.7
Standard Error	733.5131	437.6514	522.3078	384.2336	443.9396	423.466	106.2889	375.5628
Median	-50351	-47248	-45014	-44895	-51475	-47840	-43484	-45697
Mode	#N/A	#N/A	#N/A	#N/A	#N/A	#N/A	#N/A	#N/A
Standard Deviation	1270.482	758.0345	904.6637	665.5121	768.9259	733.4646	184.0978	650.4939
Sample Variance	1614124	574616.3	818416.3	442906.3	591247	537970.3	33892	423142.3
Kurtosis	#DIV/0!	#DIV/0!	#DIV/0!	#DIV/0!	#DIV/0!	#DIV/0!	#DIV/0!	#DIV/0!
Skewness	0.203832	-0.32868	1.31587	-0.59316	1.647015	1.12945	1.405926	-1.221
Range	2539	1513	1735	1322	1405	1426	350	1257
Maximum	-48995	-46575	-43702	-44368	-50231	-46829	-43210	-45359
Minimum	-51534	-48088	-45437	-45690	-51636	-48255	-43560	-46616
Sum	-150880	-141911	-134153	-134953	-153342	-142924	-130254	-137672
Count	3	3	3	3	3	3	3	3
AAD	865.5556	522.8889	677.1111	470.4444	588.6667	541.5556	138.6667	483.5556
MAD	1183	673	423	527	161	415	76	338
IQR	1269.5	756.5	867.5	661	702.5	713	175	628.5

Shapiro-Wilk Test

	0% ACN no AuNP	*5% ACN no AuNP*	*15% ACN no AuNP*	*30% ACN no AuNP*
W-stat	0.998455	0.995955	0.919527	0.986486
p-value	0.924906	0.878456	0.450673	0.777477
alpha	0.05	0.05	0.05	0.05
normal	yes	yes	yes	yes

Levene's Tests

type	p-value
means	0.537304
medians	0.876463
trimmed	0.537304

p-value >0.05

homogeneous variance

ANOVA: Single Factor

DESCRIPTION *Alpha* 0.05

Group	*Count*	*Sum*	*Mean*	*Variance*	*SS*	*Std Err*	*Lower*	*Upper*
0% ACN no AuNP	3	- 150880	-50293.3	1614124	3228248.667	458.09	-51264.4	-49322.2
5% ACN no AuNP	3	- 141911	-47303.7	574616.3	1149232.667	458.09	-48274.8	-46332.6
15% ACN no AuNP	3	- 134153	-44717.7	818416.3	1636832.667	458.09	-45688.8	-43746.6
30% ACN no AuNP	3	- 134953	-44984.3	442906.3	885812.6667	458.09	-45955.4	-44013.2
0% ACN with AuNP	3	- 153342	-51114	591247	1182494	458.09	-52085.1	-50142.9
5% ACN with AuNP	3	- 142924	-47641.3	537970.3	1075940.667	458.09	-48612.4	-46670.2
15% ACN with AuNP	3	- 130254	-43418	33892	67784	458.09	-44389.1	-42446.9
30% ACN with AuNP	3	- 137672	-45890.7	423142.3	846284.6667	458.09	-46861.8	-44919.6

ANOVA

Sources	*SS*	*df*	*MS*	*F*	*P value*	*Eta-sq*	*RMSSE*	*Omega Sq*
Between Groups	154671429.6	7	22095919	35.09855	1.50819E-08	0.938859	3.420456	0.908638
Within Groups	10072630	16	629539.4					
Total	164744059.6	23	7162785					

TUKEY HSD/KRAMER *alpha* 0.05

group	*mean*	*n*	*ss*	*df*	*q-crit*
0% ACN no AuNP	-50293.33333	3	3228249		
5% ACN no AuNP	-47303.66667	3	1149233		
15% ACN no AuNP	-44717.66667	3	1636833		
30% ACN no AuNP	-44984.33333	3	885812.7		
0% ACN with AuNP	-51114	3	1182494		
5% ACN with AuNP	-47641.33333	3	1075941		
15% ACN with AuNP	-43418	3	67784		
30% ACN with AuNP	-45890.66667	3	846284.7		
		24	10072630	16	4.896

Q TEST

group 1	*group 2*	*mean*	*std err*	*q-stat*	*lower*	*upper*	*p-value*	*mean-crit*
0% ACN no AuNP	5% ACN no AuNP	2989.7	458.09	6.526374	746.858	5232.475	0.005398063	2242.809
0% ACN no AuNP	15% ACN no AuNP	5575.7	458.09	12.17155	3332.858	7818.475	4.78152E-06	2242.809
0% ACN no AuNP	30% ACN no AuNP	5309	458.09	11.58943	3066.191	7551.809	9.06475E-06	2242.809
0% ACN no AuNP	0% ACN with AuNP	820.7	458.09	1.791497	-1422.14	3063.475	0.898362284	2242.809
0% ACN no AuNP	5% ACN with AuNP	2652	458.09	5.789255	409.1913	4894.809	0.014911433	2242.809
0% ACN no AuNP	15% ACN with AuNP	6875.3	458.09	15.0087	4632.525	9118.142	2.75564E-07	2242.809
0% ACN no AuNP	30% ACN with AuNP	4402.7	458.09	9.61092	2159.858	6645.475	9.27068E-05	2242.809
5% ACN no AuNP	15% ACN no Au-NP	2586	458.09	5.645179	343.1913	4828.809	0.018173051	2242.809
5% ACN no AuNP	30% ACN no Au-NP	2319.3	458.09	5.063052	76.52464	4562.142	0.040040722	2242.809
5% ACN no AuNP	0% ACN with AuNP	3810.3	458.09	8.31787	1567.525	6053.142	0.000481061	2242.809
5% ACN no AuNP	5% ACN with AuNP	337.7	458.09	0.737119	-1905.14	2580.475	0.999335589	2242.809
5% ACN no AuNP	15% ACN with AuNP	3885.7	458.09	8.482321	1642.858	6128.475	0.00038811	2242.809
5% ACN no AuNP	30% ACN with AuNP	1413	458.09	3.084547	-829.809	3655.809	0.410161512	2242.809
15% ACN no AuNP	30% ACN no AuNP	266.7	458.09	0.582127	-1976.14	2509.475	0.999858931	2242.809
15% ACN no AuNP	0% ACN with AuNP	6396.3	458.09	13.96305	4153.525	8639.142	7.51659E-07	2242.809
15% ACN no AuNP	5% ACN with AuNP	2923.7	458.09	6.382297	680.858	5166.475	0.006583902	2242.809
15% ACN no AuNP	15% ACN with AuNP	1299.7	458.09	2.837143	-943.142	3542.475	0.507142214	2242.809
15% ACN no AuNP	30% ACN with AuNP	1173	458.09	2.560632	-1069.81	3415.809	0.622443404	2242.809
30% ACN no AuNP	0% ACN with AuNP	6129.7	458.09	13.38092	3886.858	8372.475	1.34567E-06	2242.809
30% ACN no AuNP	5% ACN with AuNP	2657	458.09	5.80017	414.1913	4899.809	0.014689313	2242.809
30% ACN no AuNP	15% ACN with AuNP	1566.3	458.09	3.41927	-676.475	3809.142	0.296336185	2242.809
30% ACN no AuNP	30% ACN with AuNP	906.3	458.09	1.978505	-1336.48	3149.142	0.845142588	2242.809
0% ACN with AuNP	5% ACN with AuNP	3472.7	458.09	7.580752	1229.858	5715.475	0.001280915	2242.809
0% ACN with AuNP	15% ACN with AuNP	7696	458.09	16.80019	5453.191	9938.809	5.54927E-08	2242.809
0% ACN with AuNP	30% ACN with AuNP	5223.3	458.09	11.40242	2980.525	7466.142	1.11794E-05	2242.809
5% ACN with AuNP	15% ACN with AuNP	4223.3	458.09	9.21944	1980.525	6466.142	0.000151052	2242.809
5% ACN with AuNP	30% ACN with AuNP	1750.7	458.09	3.821665	-492.142	3993.475	0.191132885	2242.809
15% ACN with AuNP	30% ACN with AuNP	2472.7	458.09	5.397775	229.858	4715.475	0.025480157	2242.809

Table S5. Lennard-Jones interactions between BSA and water

BSA-water	In the absence of AuNP				In the presence of AuNP			
L-J	0% ACN no AuNP	5% ACN no AuNP	15% ACN no AuNP	30% ACN no AuNP	0% ACN with AuNP	5% ACN with AuNP	15% ACN with AuNP	30% ACN with AuNP
Run 1	-2929	-1533	-1084	-1097	-2866	-1782	-999	-1239
Run 2	-2924	-1645	-1073	-1046	-2381	-1742	-805	-1137
Run 3	-3311	-1501	-1050	-963	-2556	-1636	-935	-922

Shapiro-Wilk Test

	0% ACN no Au-NP	*5% ACN no AuNP*	*15% ACN no AuNP*	*30% ACN no AuNP*	*0% ACN with AuNP*	*5% ACN with AuNP*	*15% ACN with AuNP*	*30% ACN with AuNP*
W-stat	0.759688	0.906716	0.960133	0.981345	0.974824	0.936226	0.962853	0.959365
p-value	0.021507	0.407211	0.616082	0.738328	0.695676	0.512414	0.629584	0.612351
alpha	0.05	0.05	0.05	0.05	0.05	0.05	0.05	0.05
normal	no	yes	yes	yes	yes	yes	yes	yes

Levene's Tests

type	p-value
means	0.034575
medians	0.719387
trimmed	0.034575

p-value >0.05 homogeneous variance

Kruskal-Wallis Test

	0% ACN no AuNP	5% ACN no AuNP	15% ACN no AuNP	30% ACN no AuNP	0% ACN with AuNP	5% ACN with AuNP	15% ACN with AuNP	30% ACN with AuNP	
median	-2929	-1533	-1073	-1046	-2556	-1742	-935	-1137	
rank sum	6	32	51	55	15	25	66	50	
count	3	3	3	3	3	3	3	3	24
r^2/n	12	341.3333	867	1008.333	75	208.3333	1452	833.3333	4797.3
H-stat									20.947
H-ties									20.947
df									7
p-value									0.003849
alpha									0.05
sig									yes

DUNN's TEST			alpha	0.05	0.001786
group	*R-sum*	*size*	*R-mean*	*z-crit*	*d-crit*
0% ACN no AuNP	6	3	2		
5% ACN no AuNP	32	3	10.66667		
15% ACN no AuNP	51	3	17		
30% ACN no AuNP	55	3	18.33333		
0% ACN with AuNP	15	3	5		
5% ACN with AuNP	25	3	8.333333		
15% ACN with AuNP	66	3	22		
30% ACN with AuNP	50	3	16.66667		
		24	100	1.959964	7.713048

D TEST

group 1	*group 2*	*R-mean*	*std err*	*z-stat*	*R-crit*	*p-value*	*mean-crit*
0% ACN no AuNP	5% ACN no AuNP	8.666667	5.773503	1.501111	11.31586	0.133326932	66.84642
0% ACN no AuNP	15% ACN no AuNP	15	5.773503	2.598076	11.31586	0.009374768	115.6957
0% ACN no AuNP	30% ACN no AuNP	16.33333	5.773503	2.829016	11.31586	0.004669132	125.9798
0% ACN no AuNP	0% ACN with AuNP	3	5.773503	0.519615	11.31586	0.603331772	23.13915
0% ACN no AuNP	5% ACN with AuNP	6.333333	5.773503	1.096966	11.31586	0.272656469	48.84931
0% ACN no AuNP	15% ACN with AuNP	20	5.773503	3.464102	11.31586	0.000532006	154.261
0% ACN no AuNP	30% ACN with AuNP	14.66667	5.773503	2.540341	11.31586	0.011074438	113.1247
5% ACN no AuNP	15% ACN no AuNP	6.333333	5.773503	1.096966	11.31586	0.272656469	
5% ACN no AuNP	30% ACN no AuNP	7.666667	5.773503	1.327906	11.31586	0.184209288	
5% ACN no AuNP	0% ACN with AuNP	5.666667	5.773503	0.981495	11.31586	0.326348473	
5% ACN no AuNP	*5% ACN with AuNP*	*2.33333*	*5.7735*	0.404145	11.31586	0.686105957	
5% ACN no AuNP	15% ACN with AuNP	11.33333	5.773503	1.962991	11.31586	0.04964723	
5% ACN no AuNP	30% ACN with AuNP	6	5.773503	1.03923	11.31586	0.298697556	
15% ACN no AuNP	30% ACN no AuNP	1.333333	5.773503	0.23094	11.31586	0.817361331	
15% ACN no AuNP	0% ACN with AuNP	12	5.773503	2.078461	11.31586	0.037666922	
15% ACN no AuNP	5% ACN with AuNP	8.666667	5.773503	1.501111	11.31586	0.133326932	

15% ACN no AuNP	15% ACN with AuNP	5	5.773503	0.866025	11.31586	0.386476231
15% ACN no AuNP	30% ACN with AuNP	0.333333	5.773503	0.057735	11.31586	0.953959693
30% ACN no AuNP	0% ACN with AuNP	13.33333	5.773503	2.309401	11.31586	0.020921335
30% ACN no AuNP	*5% ACN with AuNP*	*10*	*5.7735*	1.732051	11.31586	0.083264517
30% ACN no AuNP	15% ACN with AuNP	3.666667	5.773503	0.635085	11.31586	0.525372786
30% ACN no AuNP	30% ACN with AuNP	1.666667	5.773503	0.288675	11.31586	0.772829993
0% ACN with AuNP	5% ACN with AuNP	3.333333	5.773503	0.57735	11.31586	0.563702862
0% ACN with AuNP	15% ACN with AuNP	17	5.773503	2.944486	11.31586	0.003234912
0% ACN with AuNP	30% ACN with AuNP	11.66667	5.773503	2.020726	11.31586	0.043308143
5% ACN with AuNP	15% ACN with AuNP	13.66667	5.773503	2.367136	11.31586	0.017926339
5% ACN with AuNP	30% ACN with AuNP	8.333333	5.773503	1.443376	11.31586	0.148914673
15% ACN with AuNP	30% ACN with AuNP	5.333333	5.773503	0.92376	11.31586	0.355611061

Table S6. Coulomb interactions between BSA and ACN

BSA-ACN	In the absence of Au-NP			In the presence of Au-NP		
Coulomb	5%ACN no AuNP	15% no AuNP	30% ACN no AuNP	5%ACN with AuNP	15%ACN with AuNP	30% ACN with AuNP
Run 1	-1042	-1435	-1592	-801	-1752	-1487
Run 2	-1037	-1510	-1405	-780	-1695	-1375
Run 3	-1034	-1507	-1597	-874	-1533	-1659

Shapiro-Wilk Test

	5%ACN no AuNP	*15% no AuNP*	*30% ACN no AuNP*	*5%ACN with AuNP*	*15%ACN with AuNP*	*30% ACN with AuNP*
W-stat	0.979592	0.77995	0.769518	0.907435	0.928829	0.98534
p-value	0.726225	0.067481	0.043632	0.409575	0.484242	0.768188
alpha	0.05	0.05	0.05	0.05	0.05	0.05
normal	yes	yes	no	yes	yes	yes

Levene's Tests

type	p-value
means	0.059926
medians	0.608183
trimmed	0.059926

p-value >0.05 homogeneous variance

Kruskal-Wallis Test

	5%ACN no AuNP	15%ACN no AuNP	30% ACN no AuNP	5%ACN with AuNP	15%ACN with AuNP	30% ACN with AuNP	
median	-1037	-1507	-1592	-801	-1695	-1487	
rank sum	42	25	20	51	9	24	
count	3	3	3	3	3	3	18
r^2/n	588	208.3333	133.3333	867	27	192	2015.667
H-stat							13.72515
H-ties							13.72515
df							5
p-value							0.017453
alpha							0.05
sig							yes

DUNN's TEST			alpha	0.05	0.003333
group	*R-sum*	*size*	*R-mean*	*z-crit*	
5%ACN no AuNP	42	3	14		
15%ACN no AuNP	25	3	8.333333333		
30% ACN no AuNP	20	3	6.666666667		
5%ACN with AuNP	51	3	17		
15%ACN with AuNP	9	3	3		
30% ACN with AuNP	24	3	8		
		18		1.959964	

D TEST

group 1	*group 2*	*R-mean*	*std err*	*z-stat*	*R-crit*	*p-value*
5%ACN no AuNP	15%ACN no AuNP	5.666666667	4.358898944	1.300022	8.543285	0.193593
5%ACN no AuNP	30% ACN no AuNP	7.333333333	4.358898944	1.682382	8.543285	0.092495
5%ACN no AuNP	5%ACN with AuNP	3	4.358898944	0.688247	8.543285	0.491297
5%ACN no AuNP	15%ACN with AuNP	11	4.358898944	2.523573	8.543285	0.011617
5%ACN no AuNP	30% ACN with AuNP	6	4.358898944	1.376494	8.543285	0.168669
15%ACN no AuNP	30% ACN no AuNP	1.666666667	4.358898944	0.38236	8.543285	0.702195
15%ACN no AuNP	5%ACN with AuNP	8.666666667	4.358898944	1.98827	8.543285	0.046782
15%ACN no AuNP	15%ACN with AuNP	5.333333333	4.358898944	1.223551	8.543285	0.221122
15%ACN no AuNP	30% ACN with AuNP	0.333333333	4.358898944	0.076472	8.543285	0.939044
30% ACN no AuNP	5%ACN with AuNP	10.33333333	4.358898944	2.370629	8.543285	0.017758
30% ACN no AuNP	15%ACN with AuNP	3.666666667	4.358898944	0.841191	8.543285	0.400241
30% ACN no AuNP	30% ACN with AuNP	1.333333333	4.358898944	0.305888	8.543285	0.75969
5%ACN with AuNP	15%ACN with AuNP	14	4.358898944	3.21182	8.543285	0.001319
5%ACN with AuNP	30% ACN with AuNP	9	4.358898944	2.064742	8.543285	0.038947
15%ACN with AuNP	30% ACN with AuNP	5	4.358898944	1.147079	8.543285	0.251349

Table S7. Lennard-Jones interactions between BSA and ACN

BSA-ACN	In the absence of AuNP			In the presence of AuNP		
L-J	5%ACN no AuNP	15% no AuNP	30% ACN no AuNP	5%ACN with AuNP	15%ACN with AuNP	30% ACN with AuNP
Run 1	-2940	-3878	-4517	-2575	-4911	-4245
Run 2	-3007	-4208	-3678	-2438	-4638	-3886
Run 3	-3102	-4248	-4695	-2385	-4512	-4604

Shapiro-Wilk Test

	5%ACN no AuNP	*15% no AuNP*	*30% ACN no AuNP*	*5%ACN with AuNP*	*15%ACN with AuNP*	*30% ACN with AuNP*
W-stat	0.99014	0.830032	0.876569	0.938833	0.956714	1
p-value	0.810045	0.188413	0.31438	0.5227	0.599722	1
alpha	0.05	0.05	0.05	0.05	0.05	0.05
normal	yes	yes	yes	yes	yes	yes

Levene's Tests

type	*p-value*
means	0.04993
medians	0.608042
trimmed	0.04993

p-value >0.05 homogeneous variance

ANOVA: Single Factor

DESCRIPTION Alpha 0.05

Group	*Count*	*Sum*	*Mean*	*Variance*	*SS*	*Std Err*	*Lower*	*Upper*
5%ACN no AuNP	3	-9049	-3016.3	6626.3	13252.7	170.447	-3387.7	-2644.96
15%ACN no AuNP	3	-12334	-4111.3	41233.3	82466.7	170.447	-4482.7	-3739.96
30% ACN no AuNP	3	-12890	-4296.7	294982.3	589964.7	170.447	-4668.04	-3925.3
5%ACN with AuNP	3	-7398	-2466	9613	19226	170.447	-2837.37	-2094.63
15%ACN with AuNP	3	-14061	-4687	41601	83202	170.447	-5058.37	-4315.63
30% ACN with AuNP	3	-12735	-4245	128881	257762	170.447	-4616.37	-3873.63

ANOVA								
Sources	SS	df	MS	F	P value	Eta-sq	RMSSE	Omega Sq
Between Groups	11166019.61	5	2233204	25.6	5.16127E-06	0.914	2.9225	0.872445
Within Groups	1045874	12	87156.2					
Total	12211893.61	17	718346.7					

TUKEY HSD/KRAMER		alpha		alpha	0.05
group	*mean*	*n*	*ss*	*df*	*q-crit*
5%ACN no AuNP	-3016.333333	3	13252.67		
15%ACN no AuNP	-4111.333333	3	82466.67		
30% ACN no AuNP	-4296.666667	3	589964.7		
5%ACN with AuNP	-2466	3	19226		
15%ACN with AuNP	-4687	3	83202		
30% ACN with AuNP	-4245	3	257762		
		18	1045874	12	4.75

Q TEST

group 1	*group 2*	*mean*	*std err*	*q-stat*	*lower*	*upper*	*p-value*	*mean-crit*
5%ACN no AuNP	15%ACN no AuNP	1095	170.447	6.424298	285.3785	1904.622	0.00683581	809.6212
5%ACN no AuNP	30% ACN no AuNP	1280.3	170.447	7.511637	470.7118	2089.955	0.001967088	809.6215
5%ACN no AuNP	5%ACN with AuNP	550.3	170.447	3.228772	-259.288	1359.955	0.271785125	809.6215
5%ACN no AuNP	15%ACN with AuNP	1670.7	170.447	9.801699	861.0451	2480.288	0.000178084	809.6215
5%ACN no AuNP	30% ACN with AuNP	1228.7	170.447	7.208512	419.0451	2038.288	0.002766904	809.6215
15%ACN no AuNP	30% ACN no AuNP	185.3	170.447	1.087339	-624.288	994.9549	0.967843006	809.6215
15%ACN no AuNP	5%ACN with AuNP	1645.3	170.447	9.65307	835.7118	2454.955	0.000206138	809.6215
15%ACN no AuNP	15%ACN with AuNP	575.7	170.447	3.377401	-233.955	1385.288	0.233976446	809.6215
15%ACN no AuNP	30% ACN with AuNP	133.7	170.447	0.784214	-675.955	943.2882	0.992300212	809.6215
30% ACN no AuNP	5%ACN with AuNP	1830.7	170.447	10.74041	1021.045	2640.288	7.28357E-05	809.6215
30% ACN no AuNP	15%ACN with AuNP	390.3	170.447	2.290062	-419.288	1199.955	0.602329575	809.6215
30% ACN no AuNP	30% ACN with AuNP	51.7	170.447	0.303125	-757.955	861.2882	0.999918952	809.6215
5%ACN with AuNP	15%ACN with AuNP	2221	170.447	13.03047	1411.378	3030.622	1.00528E-05	809.6215
5%ACN with AuNP	30% ACN with AuNP	1779	170.4466	10.43728	969.3785	2588.622	9.66747E-05	809.6215
15%ACN with AuNP	30% ACN with AuNP	442	170.4466	2.593187	-367.622	1251.622	0.481702055	809.6215
